# Cell-based therapies for traumatic optic neuropathy: Recent advances, challenges, and perspectives

**DOI:** 10.4103/NRR.NRR-D-24-01322

**Published:** 2025-06-19

**Authors:** Yuanhui Wang, Moxin Chen, Zhimin Tang, Ping Gu

**Affiliations:** 1 Department of Ophthalmology, Shanghai Ninth People’s Hospital, Shanghai Jiao Tong University School of Medicine, Shanghai, China; 2 Shanghai Key Laboratory of Orbital Diseases and Ocular Oncology, Shanghai, China

**Keywords:** disease models, embryonic stem cells, extracellular vesicles, mesenchymal stem cells, nerve degeneration, neural stem cells, neuroprotection, optic nerve injuries, physiopathology, regenerative medicine, retinal ganglion cells, stem cell transplantation

## Abstract

Traumatic optic neuropathy is a form of optic neuropathy resulting from trauma. Its pathophysiological mechanisms involve primary and secondary injury phases, leading to progressive retinal ganglion cell loss and axonal degeneration. Contributing factors such as physical trauma, oxidative stress, neuroinflammation, and glial scar formation exacerbate disease progression and retinal ganglion cell death. Multiple forms of cell death—including apoptosis, pyroptosis, necroptosis, and ferroptosis—are involved at different disease stages. Although current treatments, such as corticosteroid therapy and surgical interventions, have limited efficacy, cell-based therapies have emerged as a promising approach that simultaneously promotes neuroprotection and retinal ganglion cell regeneration. This review summarizes recent advances in cell-based therapies for traumatic optic neuropathy. In the context of cell replacement therapy, retinal ganglion cell-like cells derived from embryonic stem cells and induced pluripotent stem cells—via chemical induction or direct reprogramming—have demonstrated the ability to integrate into the host retina and survive for weeks to months, potentially improving visual function. Mesenchymal stem cells derived from various sources, including bone marrow, umbilical cord, placenta, and adipose tissue, have been shown to enhance retinal ganglion cell survival, stimulate axonal regeneration, and support partial functional recovery. Additionally, neural stem/progenitor cells derived from human embryonic stem cells offer neuroprotective effects and function as “neuronal relays,” facilitating reconnection between damaged regions of the optic nerve and the visual pathway. Beyond direct cell transplantation, cell-derived products, such as extracellular vesicles and cell-extracted solutions, have demonstrated promising neuroprotective effects in traumatic optic neuropathy. Despite significant progress, several challenges remain, including limited integration of transplanted cells, suboptimal functional vision recovery, the need for precise timing and delivery methods, and an incomplete understanding of the role of the retinal microenvironment and glial cell activation in neuroprotection and neuroregeneration. Furthermore, studies with longer observation periods and deeper mechanistic insights into the therapeutic effects of cell-based therapies remain scarce. Two Phase I clinical trials have confirmed the safety and potential benefits of cell-based therapy for traumatic optic neuropathy, with reported improvements in visual acuity. However, further studies are needed to validate these findings and establish significant therapeutic outcomes. In conclusion, cell-based therapies hold great promise for treating traumatic optic neuropathy, but critical obstacles must be overcome to achieve functional optic nerve regeneration. Emerging bioengineering strategies, such as scaffold-based transplantation, may improve cell survival and axonal guidance. Successful clinical translation will require rigorous preclinical validation, standardized protocols, and the integration of advanced imaging techniques to optimize therapeutic efficacy.

## Introduction

Traumatic optic neuropathy (TON) is a condition caused by extrinsic forces applied to the head or periorbital region, resulting in optic nerve injury. It is most commonly associated with road traffic accidents (Sujithra et al., 2023; Ji et al., 2025), with an incidence ranging from 0.7% to 2.5% (Lee et al., 2010). The pathophysiological mechanisms underlying TON remain incompletely understood and are considered multifactorial. Key contributing processes include direct physical trauma to the optic nerve, axonal degeneration, oxidative stress, neuroinflammation, and retinal ganglion cell (RGC) death, all of which ultimately lead to irreversible vision loss (Luo et al., 2024; Zhang et al., 2025). Despite advances in elucidating these mechanisms, the clinical management of TON remains a major challenge, underscoring the urgent need for more effective and targeted therapeutic strategies.

**Figure 1:**
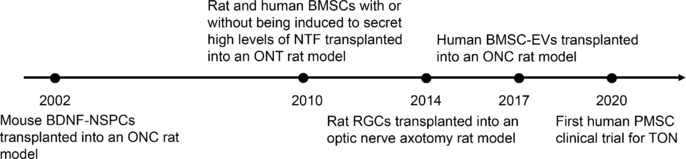
Major milestones in the development of cell-based therapy for TON. Over the past 20 years, significant advancements have been made in the use of RGC-like cells, mesenchymal stem cells, NSPCs, and other cell-based therapies for the treatment of TON. Various animal models of TON have been used to evaluate the efficacy of these therapies. Notably, Sung conducted the first clinical trial in 2020, which involved four patients and included a 12-month follow-up. No adverse effects or complications were reported during the study. BDNF-NSPC: Genetically modified neural stem/progenitor cells producing brain-derived neurotrophic factor; BMSC: bone marrow-derived mesenchymal stem cell; EV: extracellular vesicle; NSPC: neural stem/progenitor cells; NTF: neurotrophic factor; ONC: optic nerve crush; ONT: optic nerve transection; PMSC: placenta-derived mesenchymal stem cell; RGC: retinal ganglion cell; TON: traumatic optic neuropathy.

Stem cells are ubiquitously present in human and animal tissues and organs, exhibiting remarkable capacities for indefinite self-renewal and differentiation into multiple cell lineages. Cell-based therapy has emerged as a promising strategy for treating corneal blindness, macular degeneration, and retinitis pigmentosa, with numerous clinical trials currently underway worldwide (Kitazawa et al., 2022; Van Gelder et al., 2022). More recently, cell-based therapy has been proposed as a potential treatment for TON. Stem cells investigated in TON research can be classified based on their origins and characteristics into embryonic stem cells (ESCs), induced pluripotent stem cells (iPSCs), and adult stem cells, which include mesenchymal stem cells (MSCs) and neural stem/progenitor cells (NSPCs) (**Figure 1**). The optic nerve, composed of axons from approximately 1.2 million RGCs, is widely regarded as an ideal model for studying central nervous system injuries. However, RGCs in the adult mammalian retina lack regenerative capacity. Their ability to extend axons declines with age, and they lose the ability to regenerate axons early in development (Goldberg et al., 2002). Building upon these findings, various types of stem cells have been induced to differentiate into RGC-like cells as a potential replacement therapy aimed at restoring vision loss associated with RGC death. Additionally, stem cells—particularly MSCs—have demonstrated neuroprotective and immunoregulatory effects on injured RGCs by secreting neurotrophic factors and promoting RGC survival and axon regeneration, a process referred to as rescue therapy. Beyond direct stem cell transplantation, extracellular vesicles (EVs) and cell-derived extracts from stem cells, Schwann cells, and RGCs have also shown therapeutic potential in the treatment of TON (Wang et al., 2021; Zhu et al., 2023; Li et al., 2025). Notably, several clinical trials investigating stem cell-based therapies for TON are currently underway (Sung et al., 2020; Li et al., 2021).

In this review, we provide a comprehensive overview of TON, with a primary focus on its pathophysiological mechanisms and currently available rodent models. Our objective is to establish a clear framework and valuable reference for researchers and clinicians. Additionally, we examine experimental and clinical studies on cell-based therapies for TON, including the transplantation of stem cell-derived RGC-like cells, MSCs, NSPCs, and cell-derived products. Finally, we discuss the challenges and future directions of cell-based therapies for TON, with the aim of advancing both fundamental research and clinical translation in this field.

## Literature Retrieval Strategy

We conducted a comprehensive literature search in two major scientific databases, PubMed and Web of Science, to identify relevant studies for this review. The search focused on articles published between 2000 and 2025, ensuring that only the most current and pertinent studies were considered. To facilitate a thorough exploration of the topic, we used a variety of search terms, which were combined in different ways. These terms included: (traumatic optic neuropathy OR optic nerve injury OR optic nerve crush OR optic nerve transection OR optic nerve axotomy) AND ((stem cell transplantation OR cell-based therapy OR cell therapy OR retinal ganglion cell neuroprotection OR retinal ganglion cell regeneration OR exosomes OR extracellular vesicles OR retinal organoids) OR (physiopathology OR pathophysiology OR mechanisms OR models)). We carefully selected the titles, abstracts, and methods of the identified studies to ensure their relevance to the topic of cell-based therapy for TON and its underlying pathophysiological mechanisms. Only studies and review articles published in English were considered for inclusion. We excluded conference abstracts, publications lacking full-text availability, low-quality studies, and duplicate publications. In addition to preclinical studies, we also retrieved clinical trials related to the subject matter from the ClinicalTrials.gov and ChiCTR.org databases to provide a broader view of the research landscape. The literature retrieval was meticulously conducted by the author YW between May 2024 and January 2025. After a thorough screening process, a total of 189 relevant articles were included in this review.

## Traumatic Optic Neuropathy

### Epidemiology and etiology

TON is a disorder resulting from extrinsic forces applied to the craniomaxillofacial region, leading to optic nerve injury and vision impairment. The reported incidence of TON ranges from 0.7% to 2.5% (Lee et al., 2010). Recent studies have identified road traffic accidents, falls, and assaults as the most common causes of TON (Kumar et al., 2022; Wagh and Tidake, 2022; Sujithra et al., 2023). TON occurs in approximately 0.4% of all trauma cases and in 0.5% to 5.0% of cases involving closed head trauma (Sarkies, 2004; Pirouzmand, 2012). A recent survey conducted in the United Kingdom reported an incidence of at least 1.005 per million (Lee et al., 2010). In South Korea, among 2629 patients with orbital wall fractures, 27 were diagnosed with TON, corresponding to an incidence rate of 1.02% (Sakong et al., 2022). Unilateral TON accounts for more than 96% of cases (Huckhagel et al., 2020). A review of published cases indicates that fractures most commonly involve the lateral orbital wall, followed by the medial wall (Natarajan et al., 2022; Sakong et al., 2022; Sujithra et al., 2023).

### Classification and clinical manifestations

TON can be classified into direct and indirect subtypes based on the nature of axonal injury. Direct optic nerve injury occurs when bone fragments, orbital hemorrhage, or avulsion forces directly damage the optic nerve. This type of injury is often detectable through clinical examination and imaging techniques (Bastakis et al., 2019; Natarajan et al., 2022). In contrast, indirect TON (ITON) is more common and results from the transmission of force from the facial bone tissue to the optic nerve and retina during trauma (Mozo Cuadrado et al., 2020; Chen et al., 2022). Patients with ITON may present with a relative afferent pupillary defect, reduced visual acuity, visual field impairment, and/or color vision loss, despite normal findings on slit-lamp examination and imaging tests (Singman et al., 2016; Gupta et al., 2023).

### Physiopathology mechanisms

**Figure 2:**
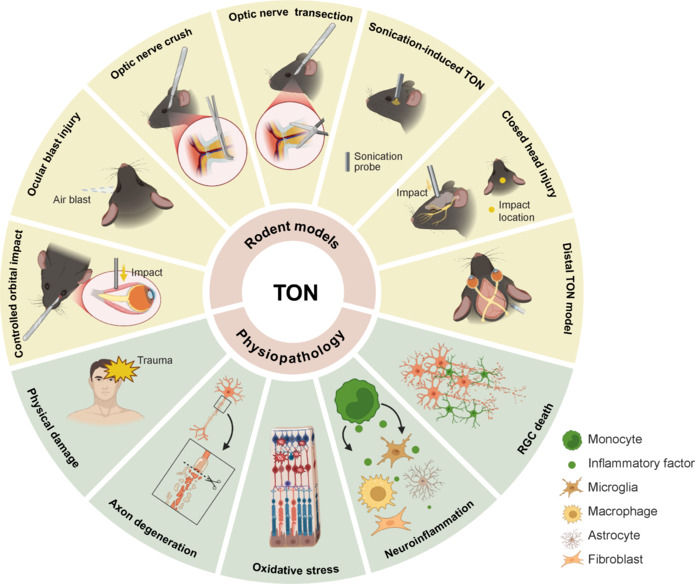
Rodent models and physiopathology of TON. This figure illustrates seven rodent models used to study TON: optic nerve crush, optic nerve transection, sonication-induced TON, closed head injury, the distal TON model, ocular blast injury, and controlled orbital impact models. Among these, the optic nerve crush model is the most widely utilized in TON research. The pathophysiological mechanisms involved in TON include physical damage, axonal degeneration, oxidative stress, neuroinflammation, and retinal ganglion cell death, with RGC death and axonal degeneration being the primary mechanisms. Created with BioRender.com. RGC: Retinal ganglion cell; TON: traumatic optic neuropathy.

The precise pathophysiological mechanisms underlying TON remain unclear, although the condition is generally considered multifactorial. TON progresses through two distinct phases: primary and secondary injury. The primary injury is characterized by direct physical trauma and anatomical disruption of the optic nerve, while the secondary injury is driven by oxidative stress and neuroinflammation. These pathological processes contribute to mitochondrial dysfunction, endoplasmic reticulum (ER) stress, glial scar formation, and RGC death. Axonal degeneration plays a role in both phases of the injury. Current insights into TON pathology highlight the interplay of physical trauma, axonal degeneration, oxidative damage, inflammatory responses, and RGC loss in disease progression (**Figure 2**).

#### Physical damage

The optic nerve in each human eye comprises approximately 1.2 million RGC axons and can be anatomically divided into four segments: intraocular (1 mm), intraorbital (25 mm), intracanalicular (5–6 mm), and intracranial (10 mm) (Kim et al., 2021b). Among these, the intracanalicular segment is the most frequently affected in TON, accounting for 71.4% of injuries (Burke et al., 2019; Chen et al., 2022). The optic nerve dura merges with the periosteum and pial vessels within the optic canal, rendering it particularly vulnerable to external trauma (Bastakis et al., 2019). A crucial aspect of TON pathophysiology involves understanding how external traumatic forces affect cranial structures. It has been reported that an impact to the frontal region can deform the ipsilateral orbital roof near the optic foramen, leading to optic nerve injury. Trauma-induced deformation and fractures of the optic canal can cause mechanical shearing of RGC axons and disrupt vascular supply. The resulting mechanical injury and ischemia contribute to optic nerve swelling, which further compromises the anastomoses between the dura and pia within the optic canal. In this confined space, swelling can lead to compartment syndrome, exacerbating ischemia in surviving RGCs and worsening optic nerve damage (Karimi et al., 2021; Chen et al., 2022).

#### Axonal degeneration

Axonal degeneration is a key pathological mechanism in TON. Following head trauma, inertial impact forces can result in diffuse axonal damage, leading to immediate deformation of axons in the brain’s white matter. This deformation disrupts the axonal cytoskeleton and impairs axoplasmic transport (Karimi et al., 2021). Subsequent calcium influx into damaged axons induces swelling, which can further contribute to secondary dysfunction, axonal breakage, and additional pathological changes in the brain (Singman et al., 2016). Axonal dysfunction impairs the retrograde transport of neurotrophic factors to RGC somas, ultimately triggering RGC apoptosis (Liu et al., 2020b). Notably, axonal dysfunction has been observed as early as 7 days after ocular blast injury (Bernardo-Colón et al., 2019). The axons extending from RGC somas converge to form the optic nerve, serving as the sole conduit for visual information transmission from the eye to the brain (Williams et al., 2020). Consequently, significant RGC loss and axonal disruption lead to irreversible vision impairment in patients with TON (Au and Ma, 2022). Furthermore, as part of the adult mammalian central nervous system, RGCs exhibit a declining capacity for axonal regeneration with age, and their ability to regenerate damaged axons is often lost even during early development (Boia et al., 2020). As a result, visual function recovery in TON patients remains limited, even when some RGCs survive the initial trauma.

Optic nerve injury disrupts the connection between axons and the brain, resulting in degeneration in two directions: anterograde degeneration (Wallerian degeneration), which occurs toward the axon terminal, and retrograde degeneration, which occurs toward the cell body. Histological analyses indicate that axonal degeneration typically begins 3–6 weeks after TON (Sung et al., 2022). Longitudinal studies have monitored changes in retinal nerve fiber layer (RNFL) thickness and the ganglion cell complex (GCC) in both animal models and humans using optical coherence tomography (Kanamori et al., 2012; Munguba et al., 2014; Sung et al., 2022). The macular RGC-inner plexiform layer (GCIPL) scan includes the RGC layer and inner plexiform layer, representing the RGC soma and dendrites in the macula lutea. In contrast, the circumpapillary RNFL (cpRNFL) represents the RGC axons as they converge at the optic disc. Studies have shown that both the GCIPL and cpRNFL begin to thin within 2 weeks after TON, while central macular thickness starts to decline after 4 weeks (Kanamori et al., 2012; Chan et al., 2019; Sung et al., 2022). Notably, changes in macular GCIPL thickness occur earlier than those in cpRNFL, suggesting that loss of RGC soma and dendrites precedes axonal degeneration in TON (Kanamori et al., 2012; Munguba et al., 2014; Sung et al., 2022). Furthermore, the reduction in central macular thickness and cpRNFL appears to stabilize around 20 weeks post-trauma, indicating that treatment interventions should be applied during this critical period (Kanamori et al., 2012; Bastakis et al., 2019).

#### Oxidative stress

Oxidative stress has been recognized as a key factor contributing to secondary injury in TON, leading to significant alterations in mitochondrial structure and function. Studies have reported elevated levels of reactive oxygen species (ROS) and biomarkers, such as calcium influx and oxidative proteins, alongside decreased levels of antioxidant enzymes like superoxide dismutase, indicating the presence of oxidative stress in TON (Kang et al., 2021; Ryan et al., 2023). Notably, ROS levels increase in the retinal ganglion cell layer (GCL) following optic nerve crush (ONC), particularly 3 days post-trauma (Wu et al., 2024). Optic nerve injury can induce mitochondrial dysfunction by disrupting mitochondrial redox homeostasis. This dysfunction results in overproduction of ROS and alterations in ROS-mediated gene expression. The impaired cellular ability to neutralize increasing ROS levels with natural antioxidants leads to progressive neurological degeneration and eventual cell death (Ryan et al., 2023). The rise in oxidative stress is also associated with calcium overload in mitochondria, which contributes to mitochondrial dysfunction and disrupts iron homeostasis, further exacerbating oxidative stress. If this cycle remains unchecked, a self-perpetuating ROS-iron-calcium loop can continuously impair neuronal and mitochondrial function, ultimately culminating in apoptosis or ferroptosis (Núñez and Hidalgo, 2019). In a study investigating the transplantation of purified rat liver mitochondria into the vitreous body of a rat ONC model, the treatment was found to promote neuroprotection of RGCs and increase the number of surviving optic nerve axons (Nascimento-Dos-Santos et al., 2020b).

When protein homeostasis is disrupted, the accumulation of misfolded and unfolded proteins in the ER can lead to ER stress, also known as the adaptive unfolded protein response (Chen et al., 2023). Protein folding is a highly redox-dependent process, and the interplay between oxidative stress and ER stress involves overlapping effector proteins, calcium flux, and shared signaling pathways (Cansler and Evanson, 2020). Several markers of ER stress have been found to increase in animal models of TON, suggesting that ER stress plays a significant role in the pathogenesis of TON (Hetzer et al., 2021).

#### Neuroinflammation

Following optic nerve injury, the neuroinflammatory response can be divided into three distinct phases: 1) Phase I, occurring within the first 3 days post-injury, involves the activation of an initial inflammatory response that aids in the clearance of cellular debris. 2) Phase II, spanning from 3 to 10 days post-injury, is characterized by the expression and release of pro-inflammatory factors by monocytes. This phase leads to the formation of scar tissue, which involves the accumulation of reactive glial cells, migration of fibroblasts, and the synthesis of extracellular matrix components. 3) Phase III begins after 10 days post-injury and can last for months. During this phase, tissue remodeling and the resolution of inflammation occur. A large number of microglia and macrophages persist at the lesion site, playing a pivotal role in enclosing the scar core, contributing to the stabilization of the injury site, and fostering a chronic inflammatory environment (Liu et al., 2023). Inflammation is essential after injury, as it modulates the local environment and influences injury progression, directly affecting the intrinsic regenerative properties of neurons (Mesquida-Veny et al., 2021). However, sterile inflammation mediated by inflammasomes such as Nlrp1b and Nlrp3 may contribute to the reduction in RGC numbers following optic nerve injury (Qijun et al., 2019). Additionally, persistent and unresolved neuroinflammation in the retina can compromise the blood–retinal barrier’s permeability after ONC, leading to sustained inflammatory reactions (Au and Ma, 2022).

Scar formation is a key aspect of the secondary injury process following initial trauma in TON. The scar at the injury site consists of three primary components: 1) a glial scar, composed of reactive astrocytes, microglia, and glial precursor cells; 2) fibrotic elements, including perivascular fibroblasts and retinal pericytes; and 3) extracellular matrix components, such as fibronectin, laminin, CD68^+^ monocytes, and ionized calcium-binding adapter molecule 1 (Iba1)-positive hematogenous macrophages (Liu et al., 2021; Jin et al., 2022). The glial scar serves a crucial role in tissue repair by containing inflammation and isolating healthy tissue from the pathological areas following injury. However, the persistence of scarring beyond the acute phase can hinder axonal regeneration and obstruct long-term recovery (Au and Ma, 2022). Chronic glial scarring has been shown to drive resident microglia and infiltrating macrophages toward a neurotoxic M1-like phenotype, exacerbating axonal damage through myelin degradation (Bradbury and Burnside, 2019). A recent study demonstrated that depleting hematogenous macrophages reduced fibrotic scar formation and preserved the normal waveform of pattern electroretinography two months after injury. This suggests that hematogenous macrophage depletion may help protect RGC function (Liu et al., 2024).

#### Retinal ganglion cell death

The proportion of RGC death varies across different TON rodent models. In ONC models, RGC loss begins 1–3 days after injury (Yang et al., 2020). Within 2–3 weeks following ONC, approximately 50% of RGCs die, which is preceded by a reduction in visual function (Liu et al., 2020b). In contrast, after ocular blast injury, RGCs decrease rapidly within 2 days post-blast (Bernardo-Colón et al., 2019). Different cell death pathways are activated at various stages following ONC. Between 3 and 14 days post-injury, apoptosis, pyroptosis, and autolysis are observed, with corresponding increases in mRNA and protein expression levels. Pyroptosis may play a leading role during the subacute cell death phase. Necroptosis, a form of regulated necrosis, is mediated by the phosphorylation of receptor-interacting serine/threonine kinase 1 (RIPK1) and receptor interacting serine/threonine kinase 3 (RIPK3) (Wu et al., 2014). Ripk3 mRNA peaks on Day 3 post-ONC, although another study reported no significant increase in Ripk3 protein expression over the 14-day period following ONC (Yao et al., 2022). Additionally, studies have demonstrated that ferroptosis contributes to RGC death in ONC and is progressively activated after injury. The ferroptosis inhibitor ferrostatin-1 effectively preserves RGC survival, retinal structure, and visual function (Guo et al., 2022; Yao et al., 2022). Morphologically, changes in RGC dendritic structure become evident 3 days after optic nerve transection, with a significant decline in the mean peak number of intersections and area under the curve over 2 weeks. This suggests a rapid and marked reduction in both the complexity and branching of RGC dendritic arbors, likely resulting from extensive pruning and retraction. These changes may lead to a loss of synaptic connections, potentially impairing the functional integrity of RGCs (Henderson et al., 2021). Furthermore, different subtypes of RGCs exhibit varying susceptibilities to optic nerve injury (Tran et al., 2019; Yang et al., 2020). RGCs can be classified into more than 40 subtypes based on morphological, functional, and genetic features. A recent study identified that direction-selective RGCs are the most resistant, whereas W3-RGCs are the most vulnerable to ONC (Yang et al., 2020). Additionally, the time course of RGC death varies among distinct resilience types of RGCs (Tran et al., 2019).

Interestingly, other injury pathways can trigger or contribute to RGC death, suggesting that RGC loss is a downstream effect in the pathogenesis of TON. For instance, axonal degeneration promotes RGC apoptosis due to a deficiency of neurotrophic factors caused by the disruption of retrograde transport (Liu et al., 2020b). Additionally, oxidative damage from inflammatory cells can induce RGC apoptotic death (Kang et al., 2021). The interactions and detailed mechanisms between these various pathways in the pathological processes of TON require further investigation.

### Rodent models

Several rodent models have been established to study TON, including optic nerve transection (axotomy), ONC, ocular blast injury, sonication-induced TON (SI-TON), closed head injury (CHI), controlled orbital impact (COI), and the distal TON model (Bastakis et al., 2019; Burke et al., 2019; Shen et al., 2023; **Figure 2**).

The standard optic nerve transection procedure involves making an incision in the scalp to expose the orbit, followed by careful dissection and retraction of the ocular tissues. The optic nerve is then precisely transected to induce a controlled injury. After transection, RGC death occurs in a progressive and specific manner, without affecting other retinal cell types (Kielczewski et al., 2005). Approximately 90% of RGC apoptosis occurs within 14 days post-transection, making this model a commonly used approach to investigate RGC apoptosis and survival (Magharious et al., 2011).

The ONC procedure involves using clips or forceps to crush the optic nerve for varying durations, such as 5, 10, 15, or up to 60 seconds. The Yasargil aneurysm clip has been shown to be an effective and reliable tool for standardizing ONC injury in experimental models (Feng et al., 2010). This method is milder than optic nerve transection and does not disrupt ocular blood flow when the optic nerve is compressed with micro-forceps for 10 seconds (Templeton and Geisert, 2012). Since the ophthalmic artery joins the optic nerve just before the lamina in mice, varying degrees of pressure applied to the optic nerve can influence retinal blood supply, thereby affecting subsequent pathological changes. Non-self-closing forceps, which progressively reduce blood flow to the optic nerve, are more appropriate for inducing ischemic injury (Shin et al., 2023). In contrast, self-closing forceps, which cause more immediate compression, are better suited for creating crush injuries. One study refers to the model created using non-self-closing forceps as “optic nerve compression” (Shin et al., 2023). However, upon reviewing recent literature on stem cell transplantation for TON, we found that the term “optic nerve compression” used in these studies (Park et al., 2018, 2021; Kwon et al., 2020) is not strictly consistent with this definition. Therefore, in this review, we have standardized the terminology and will use “ONC” to refer to the model involving the use of clips or forceps to crush the optic nerve, regardless of whether the forceps are self-closing. This model is typically used to explore the degeneration of RGCs and axon regeneration. Only a small number of RGCs undergo cell death within the first 3 days following ONC. Approximately 70% degenerate over the subsequent 5 days, with the survival rate gradually declining to around 20% by day 14 and further to approximately 10% by day 28 post-ONC (Tran et al., 2019). At 7 days following ONC for durations of 10, 15, 20, or 25 seconds, the survival rates of RGCs were approximately 47.1%, 36.4%, 32.0%, and 28.4%, respectively, compared to the naive control group (Huang et al., 2019). Notably, about 68% of the reduction in RGC soma density occurs within the first 7 days post-injury (Munguba et al., 2014). Regarding electrophysiological changes, a significant decrease in the amplitude of the positive scotopic threshold response is observed by day 3 following ONC, with alterations in the pattern electroretinogram (ERG) occurring even earlier (Liu et al., 2014; Yukita et al., 2015). Additionally, differences in the pattern ERG response and RGC death rates are observed between the BALB/cJ and C57BL/6J mouse strains, suggesting strain-specific variations in response to optic nerve injury (Templeton et al., 2009; Liu et al., 2014).

The ocular blast injury model uses a specially designed apparatus to deliver air blast waves of varying intensities to the mouse eye, inducing ocular injury. This model mirrors the characteristics of ocular blast trauma observed in patients, leading to severe anterior and posterior segment ocular injuries (Hines-Beard et al., 2012; Bricker-Anthony et al., 2014). It is primarily used to investigate ITON and exhibits distinct neuropathological features compared to direct TON (Bernardo-Colón et al., 2019). The model demonstrates high mortality rates, ranging from 24% to 46%, depending on the blast pressure, which varies between 23.5 and 30.4 pounds per square inch (psi) (Hines-Beard et al., 2012). A recent study showed that administering a 15-psi air blast twice daily for three consecutive days resulted in more pronounced axonal damage and greater visual dysfunction compared to a single 26-psi blast (Vest et al., 2019). This suggests that lower pressure combined with higher-frequency exposure may be a more effective approach. Additionally, when using the contralateral eye as a control group, it is essential to minimize physical contact between the contralateral eye and the housing chamber of the blast device to avoid unintended injury (Bricker-Anthony and Rex, 2015).

In the SI-TON model, a microtip probe sonifier is positioned directly above the optic nerve’s entry point into the bony canal, delivering an ultrasonic pulse to induce a non-contact concussive injury that results in ITON. This model minimizes confounding factors such as tissue ultrastructure destruction and surgical variability, leading to low mortality and no ocular morbidity. However, the scatter of sonication energy can cause contralateral optic nerve injury and RGC loss, preventing the use of contralateral eyes as a control group (Tao et al., 2017). The CHI model employs a closed-head weight-drop method to induce focal brain injury, with injury severity modulated by adjusting the height and weight of the falling mass. This model is commonly used to investigate mechanisms underlying human traumatic brain injury, similar to the controlled cortical impact model (Flierl et al., 2009). A recent study using the CHI model suggests that traumatic forces may be transmitted through the skull bones to the optic nerve, inducing ITON. This is supported by observations that, following skull impact, the optic canal diameter decreases, accompanied by neuroinflammation and microglial activation in the optic tract (Evanson et al., 2018). Another study utilized an electromagnetic-controlled impact device to precisely adjust the strike velocity and depth applied to the skull. They found that five consecutive mild impacts, with a strike depth of 1.0 mm and a velocity of 5 m/s, administered with an inter-concussion interval of 48 hours, induced consistent and sustained vision loss over a 10-week period and RGC loss within 6 weeks. Notably, RGC and vision loss occurred equally and simultaneously in both eyes, a pattern that contrasts with the SI-TON model (Khan et al., 2021). Similarly, the COI model uses a device adapted from the controlled cortical impact model. The blunt tip of this device strikes the extraocular tissues, which have been exposed during prior surgery, without making contact with the supraorbital ridge bone. This allows for the induction of quantifiable and manageable optic nerve injury by regulating the velocity, duration, and depth of the impact. The model has a low injury-related mortality rate and minimal ocular comorbidity. Furthermore, it avoids transmitting forces to the supraorbital ridge bone, as evidenced by ERG showing no significant difference between the contralateral eye of the injured mice and the control group, thus permitting the contralateral eyes to serve as internal controls (Ibrahim et al., 2018).

The distal TON model was recently developed using a three-dimensional (3D) stereotaxic apparatus to precisely target the cutaneous projection area of the optic canal and the intracranial segment of the optic nerve. A 27G needle is then used to puncture the distal optic nerve. RGC loss and RNFL atrophy have been confirmed in this model. This approach addresses a limitation observed in previous models, where optic nerve injury was confined to the intraorbital segment, also known as the proximal optic nerve, and did not induce damage to the distal optic nerve. However, this method is not applicable to mice with optic nerve deviation and requires the use of micro-computed tomography and flash visual evoked potential to validate the modeling outcomes (Shen et al., 2023).

Overall, the first two models are designed to study direct TON and are relatively invasive, while the remaining models are used to investigate ITON. Among these, the COI and distal TON models are minimally invasive. Each model targets different pathological aspects of TON, enabling researchers to choose the most suitable model based on their specific research objectives.

### Treatment

The clinical management of TON remains controversial, with no widely accepted guidelines. Generally, there are three major therapeutic options for patients: steroid treatment, surgery, and a combination of both approaches. Regarding steroid treatment, a recent study indicated that patients treated with high-dose corticosteroids had better visual outcomes compared to untreated patients, regardless of treatment timing (Sujithra et al., 2023). This finding aligns with previous studies (Sitaula et al., 2018; Natarajan et al., 2022). Muhafiz and Demir (2024) reported a case of TON caused by high-pressure sound waves in which the patient made a full recovery following steroid treatment. However, two studies showed no significant differences between observation alone and steroid treatment (Karimi et al., 2021; Kumar K V et al., 2022). Regarding surgery, several studies have demonstrated that decompression of the optic nerve, particularly through endoscopic transnasal optic canal decompression, is an effective treatment for TON (Sun et al., 2021; Wladis et al., 2021; Zhao et al., 2022). Patients who undergo surgery earlier, have higher preoperative visual acuity, and experience secondary vision loss may benefit more from the procedure (Huang et al., 2020; Zhao et al., 2022). When compared to steroid treatment alone, combining steroids with surgery has shown greater efficacy in treating TON. Additionally, timely surgical intervention for newly onset ITON patients can yield better outcomes (Yu et al., 2020). A recent meta-analysis indicated that conservative therapy, including observation and basic supportive care, may also be suitable for certain patients (Fallahzadeh et al., 2025). In summary, these three treatment options have been reported to aid in vision recovery for patients with TON, but clinicians have yet to reach a consensus on their overall effectiveness (Stunkel and Van Stavern, 2018; Wladis et al., 2021; Fallahzadeh et al., 2025). Therefore, there is an urgent need for more efficient and tailored therapeutics for TON.

## Cell-Based Therapy for Traumatic Optic Neuropathy

Current cell sources for cell-based therapy in TON primarily include ESCs/iPSC-derived RGC-like cells, MSCs, and NSPCs. Additionally, cell-derived products such as EVs and cellular factors have also been explored for the treatment of TON. The first study on cell-based therapy for TON was conducted in 2002, using genetically modified NSPCs that produce brain-derived neurotrophic factor (BDNF), which were transplanted into an ONC model (Wang et al., 2002). Since then, various cell types and cell-derived products have been employed in preclinical studies (Levkovitch-Verbin et al., 2010; Hertz et al., 2014; Mead and Tomarev, 2017), with the first clinical trial taking place in 2020 (Sung et al., 2020; **Figure 1**).

### Embryonic stem cells/induced pluripotent stem cells-derived retinal ganglion cell-like cells

Since RGCs lack the capacity for regeneration in the adult mammalian retina, replacing lost RGCs through transplantation presents a promising approach for cell replacement therapy. However, stem cells directly transplanted into the host eye do not spontaneously differentiate into RGCs. Instead, they tend to differentiate into cells expressing the oligodendrocyte precursor cell marker oligodendrocyte transcription factor 2 (Fu et al., 2014). Therefore, obtaining a reliable and sufficient source of RGC-like cells for replacement therapy is critical. Stem cell-derived RGC-like cells are typically generated through chemical induction or the direct reprogramming of ESCs and iPSCs. Two primary methods are employed to induce the differentiation of stem cells into RGC-like cells: 1) planar differentiation and 2) 3D differentiation (organoids). Various RGC markers, such as brain-specific homeobox/POU domain protein 3a (BRN3A), brain-specific homeobox/POU domain protein 3b (BRN3B), ISL LIM homeobox (ISL1), RNA-binding protein with multiple splicing (RBPMS), Thy-1 cell surface antigen (THY1), C–X–C motif chemokine receptor 4 (CXCR4/CD184), and L1 cell adhesion molecule (L1CAM/CD171), are commonly used to identify differentiated RGC-like cells (**Table 1**). In the next section, we will focus on the sources, generation, and transplantation of stem cell-derived RGCs in the treatment of TON.

**Table 1:** Transplantation of ESC/iPSC-derived RGC-like cells for TON treatment

Cell type	Species	Differentiation method	Differentiation period	Efficiency	Marker	Model	Administration	Dosage/eye	Observation period	Conclusion	Reference
iPSC	Human	mTeSR1 medium with Blebbistatin to form embryoid bodies; neural induction and retinal differentiation medium with heparin, B27	40–60 d	NA	CD184, CD171, BRN3B and RBPMS	ONC	Intravitreal injection	1 × 10^4^ cells	3 wk	CD184^+^CD171^+^ RGC-like cells could survive for 3 wk, and integrate into healthy and diseased retina.	Li and Luo, 2024
iPSC	Human	Overexpression of ATOH7, BRN3B, and SOX4	15–20 d	89.50%	ATOH7, BRN3A, BRN3B, EBF1, ISL1, RBPMS, SOX4, and SOX11	NA	NA	NA	NA	Establishment of an efficient method for generating human RGCs from hiPSCs through forced expression of reprogramming factors ATOH7, BRN3B, and SOX4.	Liou et al., 2023
ESC, iPSC	Human	Overexpression of NGN2	Less than 2 wk	NA	BRN3A, ISL1, SNCG, ELAVL4 (HuD), NEFL	ONC	Intravitreal injection	1 × 10^4^ cells	4 wk	iRGCs show transcriptomic profiles similar to 2-mon-old fetal or retinal organoid-derived RGCs. Demonstrated survival and integration in normal rats, mice, and ONC model.	Luo et al., 2022
iPSC	Human	MEF-conditioned media with 2-ME, B27, N2, XAV939 (X), SB431542 (SB), LDN193189 (L), nicotinamide, and IGF1; RPC induction media with X, SB, L, IGF1, and bFGF; RGC induction media with Shh and FGF8; RGCs induction media with, Follistatin 300, cyclopamine, DAPT, and Y-27632 dihydrochloride; RGC induction media with forskolin, cAMP, BDNF, NT4, CNTF; RGC induction media with DAPT and Rock inhibitor (Y27632)	36 d	87% positive of BRN3	BRN3, SNCG, THY1, RBPMS	Wild-type C57BL/6J mice	Intravitreal injection	5 × 10^5^ cells	5 mon	Intravitreal injection of hiPSC-derived RGCs in C57BL/6J mice resulted in a 94% transplantation success rate.	Vrathasha et al., 2022
ESC- derived RPC	Human	Defined medium with KSR, bFGF, EGF, L-ascorbic acid, and Y27632; differentiation medium with B27, 3-isobutyl-1-methylxanthine inhibitor, BDNF, CNTF and Rock inhibitor (Y27632)	7 d	96%	MAP2, BRN3A, THY1	NA	NA	NA	NA	A simple protocol for generating robust, homogeneous RGC-like cells from hESC-derived RPCs in 7 d to aligned PGS/PCL scaffolds provide physical environment and structural support for RGC-like cells.	Behtaj et al., 2021
Fibroblast	Human	Overexpression of ASCL1, BRN3B, and ISL1	7 d	80%	TUJ1, BRN3A	NA	NA	NA	NA	The ASCL1/BRN3B/ISL1 transcription factor combination efficiently reprograms fibroblasts into functional RGC-like neurons.	Wang et al., 2020
BMSC	Rat	Co-culture with neonatal rat retinal cells	7 d	NA	Nestin, neurofilament, Map2, and Thy1.1	NA	NA	NA	NA	Induced cells show protein profiles similar to isolated RGCs.	Xu et al., 2020
iPSC	Human	Essential 6™ medium with N2; proB27 medium with and without FGF2; retinal differentiation medium with B27	56 d	NA	THY1, BRN3A, RBPMS, HuC/D, and ISL1, TUJ1	ONC	Intravitreal injection	2 × 10^5^ cells	4 wk	Targeting THY1 is an effective strategy to select transplantable RGC-like cells	Rabesandratana et al., 2020
iPSC	Human	MEF-conditioned media with 2-ME, B27, N2, XAV939 (X), SB431542 (SB), LDN193189 (L), nicotinamide, and IGF1; RPC induction media with X, SB, L, IGF1, and bFGF; RGC induction media with Shh and FGF8; RGCs induction media with, Follistatin 300, cyclopamine, DAPT, and Y-27632 dihydrochloride; RGC induction media with forskolin, cAMP, BDNF, NT4, CNTF; RGC induction media with DAPT and Rock inhibitor (Y27632)	35 d	82%–84% positive of BRN3B	THY1, BRN3A, TUJ1, BRN3B, RBPMS, MAP2 and SNCG	NA	NA	NA	NA	Presents a reproducible and efficient method for generating high yields of RPCs from multiple iPSCs, which are then directed towards the RGC lineage	Chavali et al., 2020
ESC	Human	DMEM/F-12 medium with N2, dorsomorphin, SB-431542, and IWP2; DMEM/F-12 medium with N2, B27, bFGF, Y-27632; full satomedium with DAPT	30 d	9% positive of BRN3B, TUJ1	BRN3B, TUJ1, synaptophysin	Healthy C57BL/6 mice	Intravitreal injection	5 × 10^4^ cells	7 d	A fast consistent and low-cost protocol to generate RGC-like cells for cell replacement.	Zhang et al., 2020

This table includes various methods for generating RGC-like cells, detailing the differentiation period, efficiency, and markers used for their identification. Additionally, it presents the effects of transplanted RGC-like cells in both the ONC model and healthy models, along with information on the administration, dosage, and observation periods. 2-ME: 2-Mercaptoethanol; ASCL: achate-scute family bHLH transcription factor 1; ATOH7: atonal homolog 7;BDNF: brain-derived neurotrophic factor; bFGF: basic fibroblast growth factor; BMSC: bone marrow-derived mesenchymal stem cell; BRN3: brain-specific homeobox/POU domain protein 3; BRN3A: brain-specific homeobox/POU domain protein 3a; BRN3B: brain-specific homeobox/POU domain protein 3b; cAMP: cyclic adenosine monophosphate; CNTF: ciliary neurotrophic factor; DAPT: n-[n-(3,5-difluorophenylacetyl)-l-alanylleucine]; EBF1: early B-cell factor 1; EGF: epidermal growth factor; ELAVL4 (HuD): embryonic lethal abnormal vision-like protein 4; ESC: embryonic stem cell; FGF2: fibroblast growth factor 2; FGF8: fibroblast growth factor 8; hiPSC: human induced pluripotent stem cell; IGF1: insulin-like growth factor 1; iPSC: induced pluripotent stem cell; iRGC: induced retinal ganglion cell; ISL1: ISL LIM homeobox 1; MAP2: microtubule-associated protein 2; MEF: mouse embryonic fibroblast; NA: not applicable; NEFL: neurofilament light chain; NGN2: neurogenin 2; NT4: neurotrophin 4; ONC: optic nerve crush; PCL: poly (ε-caprolactone); PGS: poly (glycerol sebacate); RBPMS: RNA-binding protein with multiple splicing; RGC: retinal ganglion cell; Rock: Rho kinase; RPC: retinal progenitor cell; Shh: sonic hedgehog; SNCG: synuclein gamma; SOX11: SRY-box transcription factor 11; SOX4: SRY-box transcription factor 4; THY1: Thy-1 cell surface antigen; TON: traumatic optic neuropathy; TUJ1: βIII-tubulin.

#### Characteristics of embryonic stem cells and induced pluripotent stem cells

ESCs are derived from the inner cell mass of the blastocyst before gastrulation and have the ability to self-renew (Dekel et al., 2022; Varzideh et al., 2023). ESCs can differentiate into various retinal cell types, including RGCs (Behtaj et al., 2021), retinal pigment epithelial cells (Ben M’Barek et al., 2017), and photoreceptor cells (Osakada et al., 2008), providing an abundant source for replacement therapy. However, ESCs also present challenges, such as difficulties in obtaining specific cell types and limited proliferative capacity. Additionally, ethical concerns arise from the isolation of ESCs, as this process involves manipulating human fetal tissues. The potential for teratoma formation *in vivo* and the challenge of finding compatible cells for patients necessitate additional immunosuppressive treatment, increasing the treatment burden. In contrast, iPSCs are adult somatic cells reprogrammed through the overexpression of Yamanaka factors, including Oct3/4, Sox2, Klf4, and c-Myc (Takahashi et al., 2007). These cells can be derived reliably from various human sources, such as fibroblasts, keratinocytes, and hematopoietic cells, offering a broad range of potential sources for stem cell therapy (Liu et al., 2020a). iPSCs have the advantage of a low risk of immune rejection since they can be used in autologous transplantation. They exhibit chromosomal structures, gene expression profiles, and pluripotency characteristics similar to ESCs, enabling differentiation into RGCs (Guenther et al., 2010; Wong et al., 2023). However, their low differentiation efficiency may limit their applications. While iPSCs help avoid the ethical issues associated with ESCs and offer personalized cell therapy possibilities, the reprogramming process can lead to genomic instability and an increased risk of gene mutations. Additionally, some reprogramming factors, such as c-Myc, are proto-oncogenes, which may increase the risk of tumor formation.

#### Retinal ganglion cell–like cells derived from embryonic stem cells, induced pluripotent stem cells, and other cell types

The planar differentiation of RGC-like cells derived from ESCs or iPSCs typically involves a two-stage process. The first stage is the differentiation of ESCs or iPSCs into retinal progenitor cells (RPCs), followed by the maturation of RPCs into RGC-like cells in the second stage. Using chemically defined media can reduce reliance on protein growth factors. For example, one study achieved the differentiation of human ESC (hESC)-derived RGCs using a chemically defined medium in just 4 weeks, although only 9% of the resulting cells were positive for BRN3B and βIII-tubulin (TUJ1) (Zhang et al., 2020). Behtaj et al. (2021) reported that after seven days of culturing hESC-derived RPCs in RGC differentiation media containing ciliary neurotrophic factor (CNTF), BDNF, the phosphodiesterase inhibitor 3-isobutyl-1-methylxanthine, and the rho-associated coiled-coil forming protein kinase inhibitor Y27632, over 90% of the cells expressed microtubule-associated protein 2 (MAP2) and BRN3A. Moreover, more than 70% of the cells expressed THY1 and BRN3A, suggesting that this method is effective for differentiating hESC-derived RPCs into RGC-like cells. Additionally, they found that a polymeric scaffold made from aligned poly(glycerol sebacate) and poly(ε-caprolactone) provided an ideal substrate for RGC-like cells to attach and grow, offering structural support for axon extension and neurite outgrowth. A recent study also reported that novel combinations of chemical molecules could enhance the generation of RGC-like cells. For instance, the use of small molecules and peptide modulators to inhibit the bone morphogenetic protein, transforming growth factor-β, and Wnt pathways resulted in over 80% of iPSC-derived cells expressing BRN3B after 35 days. Following enrichment through CD90.2-based magnetic-activated cell sorting, the proportion of cells expressing BRN3A increased to nearly 95% (Chavali et al., 2020).

While planar differentiation has limitations in terms of multicellular organization, these can be addressed through the use of retinal organoids. Retinal organoids are 3D miniature models of the retina developed *in vitro* from stem cells, showing greater similarity to the temporal development and spatial structure of the retina, with RGC-like cells present at various stages of maturation (Zhang et al., 2021a; Li and Luo, 2024). The initial creation of mouse retinal organoids involved culturing ESCs in a 3D suspension of embryoid body aggregates, which autonomously organized into layered structures resembling the embryonic optic cup (Eiraku et al., 2011). RGC-like cells are the first to proliferate in hiPSC-derived retinal organoids, gradually increasing in number over 1 week and typically localizing to the basal layer (Ohlemacher et al., 2019; Li and Luo, 2024). One study demonstrated that the population of RGC-like cells began to increase on day 42 of differentiation, peaked around day 56, and then gradually declined (Rabesandratana et al., 2020). Interestingly, RGC-like cells were not observed beyond day 25 of differentiation in mouse retinal organoids, with degeneration starting around day 32 (Brooks et al., 2019). The timing of the peak in RGC-like cell numbers and the subsequent decline varies among different protocols for generating RGC-like cells from retinal organoids. RGC-like cells make up approximately 0.1% to 30% of the total cells in retinal organoids (Hua et al., 2020). Both planar and 3D differentiation can yield large quantities of RGC-like cells for applications; however, cells derived from 3D differentiation exhibit a greater transcriptional profile similarity to human fetal RGCs (Sridhar et al., 2020). A study collected RGC-like cells from hiPSC-derived retinal organoids on day 56, when their population peaked. After purification using THY1-based magnetic-activated cell sorting, the proportion of RGC-like cells increased from 60% under adherent culture conditions on day 56 to 80% on day 63 (Rabesandratana et al., 2020). Li and Luo (2024) performed fluorescence-activated cell sorting using the surface markers CD184 and CD171 to purify RGC-like cells from retinal organoids between days 40 and 60. They found that, on average, 21% of the cells were CD184^+^CD171^+^, exhibiting RGC-like morphological features, such as large, bright cell bodies and extended neurofilaments. These subtype RGC-like cells not only displayed the ability to proliferate but also successfully passed through multiple passages, expressing higher levels of α-type RGC markers. Moreover, in a Matrigel-based 3D environment, the CD184^+^CD171^+^ RGC-like cells demonstrated the ability to form spheroids and expressed TUJ1 and doublecortin in their cell bodies, although they did not extend axons. This suggests that the Matrigel-based 3D environment may help maintain and enhance the viability and proliferative capacity of RGC-like cells.

Additionally, transcription factor-based cellular conversion can achieve higher efficiency in generating RGC-like cells from stem cells, both in planar and 3D differentiation. A landmark study in 2022 demonstrated that the overexpression of neurogenin 2 (NGN2) alone could convert human iPSCs (hiPSCs) and hESCs into induced RGCs (iRGCs) with nearly 100% yield and purity in less than 10 days. Furthermore, these iRGCs exhibited electrophysiological responses resembling those of immature primary RGCs and showed a greater similarity to fetal RGCs at the transcriptome level compared to RGCs derived from retinal organoids (Luo et al., 2022). In addition, the overexpression of atonal homolog 7 (ATOH7), BRN3B, and SRY-box transcription factor 4 (SOX4) in hiPSCs can generate iRGCs with over 90% efficiency within 15 to 20 days (Liou et al., 2023). Moreover, the combination of three transcription factors—achaete-scute family bHLH transcription factor 1 (ASCL1), BRN3B, and ISL1—can directly reprogram fibroblasts into iRGCs, achieving 80% efficiency by day 2. These iRGCs exhibit transcriptomic profiles closely resembling those of native RGCs (Wang et al., 2020).

#### Transplantation of retinal ganglion cells derived from embryonic stem cells/induced pluripotent stem cells

The studies on the transplantation of RGCs derived from ESCs and iPSCs for treating TON are summarized in **Table 1**. hESC-derived RGC-like cells have been shown to integrate into a healthy host retina as early as 1 week after injection (Zhang et al., 2020). In a study using the ONC rat model, hiPSC-derived RGCs were intravitreally transplanted. These transplanted RGC-like cells were detected in 50% of the animals 4 weeks post-injection, either in small clusters near the retina or as solitary cells integrated within the host GCL (Rabésandratana et al., 2020). In a more recent study, the same research team intravitreally transplanted hiPSC-derived RGCs into wild-type C57BL/6J mice, achieving a successful transplantation rate of approximately 94%. The transplanted RGCs integrated within the GCL and survived for at least 5 months, exhibiting morphology and functional behaviors comparable to native murine RGCs (Vrathasha et al., 2022). Furthermore, hiPSC-derived CD184^+^CD171^+^ RGCs were intravitreally injected into the eyes of both normal adult mice and those with ONC injuries. The transplanted RGC-like cells survived for 3 weeks, migrated to the optic nerve head, and extended into the crushed optic nerve (Li and Luo, 2024). Additionally, iRGCs generated by overexpressing NGN2 in hiPSCs and hESCs survived in healthy host retinas for 4 weeks, migrating into the GCL and occasionally passing through it. These transplanted iRGCs promoted the survival of endogenous RGCs for 1 week after ONC, although this effect diminished by 2 weeks. However, in the ONC model, the survival and integration of iRGCs were lower compared to those in healthy host eyes (Luo et al., 2022). Luo et al. (2011) also demonstrated that retinal organoids, which preserve the intrinsic cell alignment of RGC-like cells, can be transplanted directly. They seeded the retinal organoids onto Matrigel-coated, degradable poly(lactic-co-glycolic acid) scaffolds and transplanted the tissue-engineered retinal sheets, containing functional RGC-like cells, into the eyes of rhesus monkeys. The RGC-like cells survived, matured further, and migrated on the surface of the host retina, with no tumorigenesis or visible damage to the host retina observed.

Müller glial cells have been shown to enhance the survival and neurogenesis of RGC-like cells derived from retinal organoids, as well as support the survival of progenitor cells that can differentiate into RGCs, by increasing the expression of Atoh7 (Pereiro et al., 2020). Additionally, astrocytes have been demonstrated to promote the morphological and functional maturation of RGC-like cells. When co-cultured with astrocytes, RGC-like cells extend longer and more complex neurites, generate repetitive action potentials, and establish presumptive synaptic contacts, mimicking the behavior of RGCs *in vivo* (VanderWall et al., 2019). However, these results have only been observed *in vitro* thus far. Interestingly, iPSCs not only enhance neurite extension and RGC survival *in vitro* but also promote these effects in the adult rat eye following intravitreal co-injection of iPSCs and RGCs (Wu et al., 2018). Therefore, co-injecting Müller glial cells, astrocytes, iPSCs, or other supportive cell types along with RGC-like cells in TON models may be a promising approach for future studies and could lead to greater vision improvement compared to transplanting RGC-like cells alone. Moreover, since iPSCs appear to undergo changes in morphology and differentiation when injected into healthy host eyes, the specific cell types they differentiate into remain uncertain and require further investigation (Wu et al., 2018). A previous study in a laser-induced glaucoma model suggested that iPSCs may differentiate into oligodendrocyte precursor cells, as evidenced by the expression of glial cell markers in the generated cells (Fu et al., 2014).

In summary, both ESCs and iPSCs offer distinct advantages and challenges. The generation of RGC-like cells from ESCs, iPSCs, or other cell types typically ranges from 7 to 60 days, with yields varying from 9% to 100%, depending on the methods and cell types used (**Table 1**). Generating RGC-like cells from retinal organoids generally takes longer compared to the direct conversion of stem cells into RGC-like cells through reprogramming and may require additional purification steps. However, recent studies indicate that cryopreserved organoids can provide RGC-like cells, regardless of whether they are dissociated, allowing for the storage of organoids at specific stages of differentiation for future applications (Rabésandratana et al., 2020; Luo et al., 2021). The process of dissociating single RGC-like cells from retinal organoids or cell cultures is crucial for ensuring a higher yield of viable and healthy RGC-like cells for transplantation. RGC-like cells can survive from 3 weeks to 5 months in both healthy host retinas and ONC models, with a tendency to integrate into the GCL (**Table 1**). Since material exchange may complicate the interpretation of findings derived from fluorescence-based tracking tools (Nickerson et al., 2018), it is notable that no material exchange between transplanted RGC-like cells and host RGCs has been observed in RGC replacement therapy for TON (Wu et al., 2018; Vrathasha et al., 2022). However, further studies are needed to confirm these results definitively. Additionally, the subtype of RGCs and the maturity of RGC-like cells may affect the success of replacement therapy. Given the differences in morphological, functional, and genetic features, as well as resilience to optic nerve injury among RGC subtypes (Tran et al., 2019; Yang et al., 2020), transplanting specific RGC subtypes tailored to particular diseases may lead to optimal therapeutic outcomes. Two studies have characterized the diversity of stem cell–derived RGC-like cells based on their molecular signatures and physiological properties (Langer et al., 2018; Gudiseva et al., 2021). Adult primary RGCs exhibit reduced survival, migration, and integration capacity compared to embryonic and postnatal primary RGCs, as well as *in vitro* differentiated RGCs (Hertz et al., 2014). The likelihood of transplanted RGCs integrating into the host retina improves when the developmental stages of the donor and recipient retinas are closely matched (Chen et al., 2010). Timing is also critical for successful transplantation. Hertz et al. (2014) found that a degenerating retina begins to support the survival of engrafted RGCs only 28 days after optic nerve transection. However, in the ONC model, 28 days seems insufficient, as the survival of engrafted RGC-like cells in ONC-treated eyes is significantly lower than in sham-treated eyes (Luo et al., 2022). While a degenerating retina is typically considered more permissive for grafted cells to migrate and integrate than a healthy retina (Chen et al., 2010; Vrathasha et al., 2022), one study suggests otherwise (Luo et al., 2022). Further research is needed to investigate the transplantation of stem cell-derived RGCs at different time points after optic nerve injury to determine the optimal timing for successful integration. Additionally, the inflammatory response triggered by the donor cells, which are xenografts, may affect the survival and integration of stem cell-derived RGCs, representing another crucial question for future exploration. Overall, further experiments involving the transplantation of stem cell-derived RGCs into animal models hold promise for advancing replacement therapy.

### Mesenchymal stem cells

#### Characteristics of mesenchymal stem cells

MSCs are one of the dominant cell sources for therapy in TON. Originally identified in bone marrow in 1974, the isolation and *in vitro* expansion of human bone marrow-derived MSCs (hBMSCs) were first documented in 1992. These cells were subsequently infused into patients in 1993, with the results reported in 1995 (Lucas-Ruiz et al., 2019; Pittenger et al., 2019). Further research has identified MSCs in various other tissues, including adipose tissue, dental tissues (such as dental pulp and periodontal ligament), and perinatal derivatives (such as umbilical cord blood, Wharton’s jelly, amniotic membrane, and placenta) (Cen et al., 2018; Hu et al., 2021; Tan et al., 2022). The International Society for Cellular Therapy defines MSCs according to the following criteria: (1) plastic adherence, (2) positive expression of CD73, CD105, and CD90 markers, and negative expression of CD45, CD14, CD34, and HLA-DR markers, and (3) the ability to differentiate into mesodermal lineages under appropriate *in vitro* conditions (Dominici et al., 2006). MSCs originate from the mesoderm and have the potential to differentiate into adipocytes, chondrocytes, and osteocytes (Holan et al., 2021). Additionally, MSCs have been shown to differentiate into neurons in the retina, including photoreceptors, retinal pigment epithelial cells, and RGCs. However, their rate of differentiation into damaged cells and migration is relatively low (Xu et al., 2020; Coco-Martin et al., 2021). MSCs also exhibit neuroprotective, immunomodulatory, antioxidant, and anti-apoptotic properties by secreting biologically active compounds such as growth factors and cytokines, as well as transferring mitochondria and utilizing other mechanisms (Hu et al., 2021; Sanie-Jahromi et al., 2022; Tan et al., 2022; Usategui-Martín et al., 2022).

Compared to ESCs, MSCs can be sourced from various tissues with minimal harm, and they can be expanded *in vitro*, thereby avoiding moral objections and ethical concerns while also enabling autologous transplantation (Hu et al., 2021). Additionally, the immunomodulatory properties of MSCs, along with their low expression of HLA-DR and minimal immunogenicity, make them suitable for both autologous and allogeneic transplantation (Lucas-Ruiz et al., 2019). The neuroprotective effects of MSCs occur naturally, without the need for reprogramming or genetic modification, and MSCs from different sources exhibit similar therapeutic effects (Holan et al., 2021). As a result, MSCs are considered a promising candidate for stem cell therapy in degenerative diseases, with most research focusing on their application in rescue therapy.

#### Transplantation of mesenchymal stem cells

Several studies have investigated the potential benefits of MSC transplantation in the treatment of TON, as well as the underlying mechanisms driving the therapeutic effects (**Table 2**).

**Table 2:** MSC transplantation for TON treatment

Cell type	Species	Administration	Model	Dosage/eye	Integration	Observation period	Molecular mechanism	Conclusion	Reference
BMSC	Human	Intravitreal injection	ONC	5 × 10^4^ cells	NA	14 d	Promote activation of STAT3; GTE increases BDNF secretion	Combination of BMSC and GTE promotes RGC survival, neurite outgrowth, axonal regeneration.	Yang et al., 2024
WJ-MSC	Human	Peribulbar injection	ONC	1 × 10^7^ cells	Yes	42 d	Potential anti-inflammatory effects	WJ-MSCs have the potential to differentiate into axonal-like cells.	Looi et al., 2022
WJ-MSC	Human	Intravitreal injection	ONC	5 × 10^5^ cells	NA	120 d	Preferentially increase the survival of α-RGCs	WJ-MSCs transplantation gives rise to long-term survival of RGCs, marked long-distance axonal regeneration, and partial recovery of synaptic function.	da Silva-Junior et al., 2021
PMSC	Human	Subtenon injection	ONC	2 × 10^6^ cells	NA	28 d	The expressions of markers for optic nerve regeneration and cell viability markers such as vimentin, THY-1, GFAP, ermin, and neurofilaments were increased by hypoxia-preconditioned PMSC.	Hypoxia-preconditioned PMSCs increase retinal RGC survival and promote optic nerve axon regeneration.	Kwon et al., 2020
UCB-MSC	Human	Intravitreal injection	ONC	2 × 10^4^ cells	NA	35 d	2D-MSCs secrete abundant signaling factors and activate the JAK/STAT3 and MAPK/ERK signaling pathways.	2D-MSCs promoted RGCs survival, optic nerve axonal regeneration, and improved visual dysfunction induced by ONC.	Huang et al., 2019
BMSC	Rat	Intravitreal injection	ONC	5 × 10^5^ cells	NA	240 d	NA	BMSCs promoted long-term neuroprotection, long-distance axon regeneration, and synaptic reconnection after ONC	Mesentier-Louro et al., 2019
WJ-MSC	Human	Intravitreal injection	ONC	2 × 10^4^ cells	Yes	30 d	Overexpression of anti-inflammatory cytokines and trophic factors	Intravitreally administered WJ-MSCs are not toxic to RGCs; instead, they are neuroprotective for RGC survival and can integrate into the GCL, surviving for at least 30 d.	Millán-Rivero et al., 2018
PLSC	Human	Intravitreal injection	ONC	5 × 10^4^ cells	NA	21 d	The mechanisms underlying the therapeutic effect are cell–cell interaction and promotion of BDNF secretion, but not improvement of endogenous progenitor cell regeneration.	PLSCs promote RGC survival and axonal regeneration in both *in vitro* and *in vivo* ONC models.	Cen et al., 2018
ADSC	Rat	Subtenon injection	ONC	1 × 10^6^ cells	NA	28 d	Downregulation of the proteins elevated in the TLR4 pathway caused by ONC.	ADSCs improve vision degeneration after ONC.	Wang et al., 2018
PMSC	Human	Intravenous injection	ONC	1 × 10^6^ cells	NA	28 d	NA	PMSCs improve axon survival rates in the ONC model.	Park et al., 2018
ADSC	Rat	Intravitreal injection	ONC	3 × 10^4^ cells	NA	28 d	The neuroprotective effect on RGCs may be associated with the inhibition of apoptosis following ONC.	ADSCs promote the survival of RGCs and axon regeneration.	Li et al., 2018
Primary MSC and Schwann cell	Rat	Intravitreal injection	ONC	5 × 10^4^ MSCs + 5 × 10^4^ Schwann cells	NA	14 d	Co-injection of MSCs and Schwann cells reduced RGC apoptosis, regulated the expression of apoptosis-related proteins, and enhanced the secretion of neurotrophic factors and axon regeneration-associated proteins.	Schwann cells combined with MSCs could promote RGC survival, and their therapeutic effect is superior to that of the individual use of Schwann cells or MSCs.	Chen et al., 2019a

An overview of various sources of MSCs used in cell-based therapy for TON, including details on administration, rodent models, dosage, and observation periods is provided. The outcomes of MSC transplantation, including potential integration with the host retina and the underlying mechanisms, are also presented. 2D-MSC: Mesenchymal stem cell cultured in 2D monolayers; ADSC: adipose-derived stem cell; BDNF: brain-derived neurotrophic factor; BMSC: bone marrow-derived mesenchymal stem cell; GCL: ganglion cell layer; GFAP: glial fibrillary acidic protein; GTE: green tea extract; JAK/STAT3: janus kinase/signal transducer and activator of transcription 3; MAPK/ERK: mitogen-activated protein kinase/extracellular signal-regulated kinase; MSC: mesenchymal stem cell; NA: not applicable; ONC: optic nerve crush; PLSC: periodontal ligament-derived stem cell; PMSC: placenta-derived mesenchymal stem cell; RGC: retinal ganglion cell; STAT3: signal transducer and activator of transcription 3; TLR4: toll-like receptor 4; TON: traumatic optic neuropathy; UCB-MSC: umbilical cord blood-derived mesenchymal stem cell; WJ-MSC: umbilical cord Wharton’s jelly-derived mesenchymal stem cell.

Rat adipose-derived stem cells have been shown to promote RGC survival and axon regeneration in the ONC model (Li et al., 2018). These cells also help improve vision impairment, as evidenced by the inhibition of P1 wave amplitude decrease and the increase in wave latency (Wang et al., 2018). Similarly, human periodontal ligament-derived stem cells exhibited comparable effects both *in vivo* in the ONC model and *in vitro*, with mechanisms involving direct cellular interactions and increased secretion of BDNF. However, they did not promote the regeneration of endogenous progenitor cells (Cen et al., 2018).

BMSCs have been shown to provide neuroprotection, enhancing RGC survival and promoting axon regeneration. This is evidenced by an increase in the number of RGCs, as well as both the number and length of axons, up to 28 days after ONC compared to the control group. This effect may be associated with the upregulation of fibroblast growth factor 2 and interleukin-1β expression in the GCL. Mesentier-Louro et al. (2014) found that the injected BMSCs primarily remained in the vitreous body for up to 18 weeks. More recently, the same research team demonstrated that the neuroprotective effect of injected BMSCs can be sustained for up to 240 days. Additionally, regenerated axons were observed to bypass the injury site via the periphery, progress through the central regions of the mid/distal nerve, and extend toward the superior colliculus, where they formed brief synaptic connections. Unfortunately, the animals exhibited limited functional recovery, as visual behaviors such as the optokinetic response and light perception did not improve (Mesentier-Louro et al., 2019). Another study found that the combination of hBMSCs and green tea extract (GTE) promoted neurite outgrowth and RGC survival both *in vitro* and in the ONC model *in vivo*, possibly due to increased activation of signal transducer and activator of transcription 3 (STAT3). Furthermore, GTE was shown to enhance the secretion of BDNF by hBMSCs *in vitro*, although this effect requires confirmation *in vivo* (Yang et al., 2024).

MSCs can be derived from umbilical cord blood, Wharton’s jelly, and bone marrow. Umbilical cord blood–derived MSCs (UCB-MSCs) and Wharton’s jelly–derived MSCs (WJ-MSCs) offer several advantages over other MSCs, primarily due to the non-invasive nature of umbilical cord tissue acquisition and the reduced epigenetic alterations compared to adult tissues. Furthermore, WJ-MSCs express a broader range of tumor suppressor factors than UCB-MSCs. Research has shown that human WJ-MSCs do not exhibit toxicity when injected intravitreally into rats with intact retinas (Millán-Rivero et al., 2018). In animal models of ONC, WJ-MSC injections promoted RGC survival, long axonal regeneration, partial recovery of synaptic function, and preservation of optic nerve function (Millán-Rivero et al., 2018; da Silva-Junior et al., 2021; Looi et al., 2022). These studies used vitreous injections at doses ranging from 2 × 10^4^ to 5 × 10^5^ cells, and peribulbar injections at a dose of 10^7^ cells. However, extensive infiltration and activation of Iba1^+^ cells in the ONC model, induced by human WJ-MSCs, disrupted retinal structure (Millán-Rivero et al., 2018). It remains uncertain whether this structural disruption affects retinal function, although it appears to be transient. The effectiveness of UCB-MSCs is also influenced by their state and microenvironment. One study found that two-dimensional (2D) UCB-MSCs exhibited stronger neuroprotective effects than spheroid UCB-MSCs, as evidenced by enhanced RGC survival and axon regeneration. Moreover, 2D UCB-MSCs significantly improved visual function, as measured by flash visual evoked potentials, whereas spheroid UCB-MSCs did not show significant improvements. Following intravitreal injection of 2D UCB-MSCs, several signaling factors, including stem cell growth factor-β, hepatocyte growth factor, and monocyte chemoattractant protein-1, were secreted, activating the Janus kinase/signal transducer and activator of transcription 3 (JAK/STAT3) and mitogen-activated protein kinase/extracellular signal-regulated kinase (MAPK/ERK) signaling pathways (Huang et al., 2019). Additionally, human placenta-derived MSCs (hPMSCs), both with and without hypoxia preconditioning, have demonstrated the ability to promote RGC survival and axon regeneration in the ONC model (Park et al., 2018; Kwon et al., 2020). Notably, hPMSCs subjected to hypoxia preconditioning exhibited enhanced neuroprotective effects compared to naive hPMSCs, potentially mediated by vascular endothelial growth factor (VEGF) (Kwon et al., 2020). Co-injection of different cell types is also a strategy used in MSC rescue therapy. For example, co-injecting Schwann cells and MSCs has shown remarkable reparative effects, including an increase in the number of RGCs and a decrease in RGC apoptosis in the ONC model. This combined approach proves to be more effective than using Schwann cells or MSCs alone, with improved outcomes likely mediated by an increase in B-cell lymphoma 2 (Bcl-2), a decrease in Bcl-2-associated X protein (Bax), and elevated expression of BDNF and growth-associated protein 43 (GAP43) (Chen et al., 2019a). Moreover, combining gene therapy with MSC therapy is an emerging and promising avenue of research. For instance, the overexpression of pigment epithelium-derived factor (PEDF) in conjunction with the transplantation of human MSCs into the ONC model has shown superior neuroprotective effects compared to *PEDF* gene therapy alone (Nascimento-Dos-Santos et al., 2020a).

Various sources of MSCs have been utilized in rescue therapy for TON, including adipose tissue, periodontal ligament, bone marrow, umbilical cord blood, Wharton’s jelly from the umbilical cord, and placenta (**Table 2**). MSCs have demonstrated neuroprotective effects lasting between 14 and 240 days following injection (**Table 2**). The long-term effects of MSCs appear to depend on both the dosage and the survival duration of the injected cells. Notably, the delivery routes for stem cells influence the required cell number and concentration. In a study reporting the longest-lasting effects, which persisted for 240 days post-injection, 5 × 10^5^ rat BMSCs were delivered via intravitreal injection (Mesentier-Louro et al., 2019). This dosage is the highest among studies using this route, with MSC survival documented for at least 18 weeks. Similarly, the second-longest duration of 120 days was achieved using the same dosage of human WJ-MSCs via intravitreal injection (da Silva-Junior et al., 2021). The survival time of MSCs varies from 2 to 18 weeks, according to studies that specifically examined their survival post-transplantation (Mesentier-Louro et al., 2014; Cen et al., 2018; Millán-Rivero et al., 2018). We hypothesize that the species origin of the transplanted cells may also influence the survival duration of MSCs, as the xenogeneic nature of human WJ-MSCs in animal models might affect how long MSCs remain viable within host tissue. Injected MSCs can induce the infiltration of inflammatory cells (Cen et al., 2018; Millán-Rivero et al., 2018; Huang et al., 2019). While previous studies suggest that activated Iba1^+^ cells, which represent microglia and macrophages, facilitate the neuroprotective effects of MSCs (Cen et al., 2007, 2018), one study has shown that these Iba1^+^ cells can temporarily disrupt the retinal layered structure (Millán-Rivero et al., 2018). Therefore, we cannot conclude that the infiltration of inflammatory cells is entirely detrimental or beneficial. MSCs have been shown to regulate inflammatory cytokines and interact with immune cells to create a more favorable environment for RGCs. For example, MSCs can increase the expression of interleukin-1β in the host retina and inhibit the upregulation of inflammatory factors in the toll-like receptor 4 signaling pathway (Mesentier-Louro et al., 2014; Wang et al., 2018). Moreover, MSCs can decrease microglia survival and alter the phenotype of microglia in *in vitro* retinal explants (Teixeira-Pinheiro et al., 2020). When MSCs are transplanted into the vitreous, most remain within the vitreous cavity, while a small proportion migrate into the GCL (Mesentier-Louro et al., 2014; Millán-Rivero et al., 2018; Huang et al., 2019). One study reported that transplanted MSCs have the potential to differentiate into glial tissue, including astrocytes; however, other studies have not observed such differentiation (Yu et al., 2006; Huang et al., 2019; Looi et al., 2022). GTE suppresses MSC proliferation and differentiation while enhancing their neuroprotective functions (Yang et al., 2024). These findings suggest that MSC differentiation is not necessary for their neuroprotective effects. The properties and behaviors of MSCs can be influenced by culture conditions. For instance, hypoxic preconditioning can enhance MSC functions, including their neuroprotective and pro-regenerative capacities (Kwon et al., 2020). Additionally, whether MSCs are cultured in 2D monolayers or 3D spheroids, as well as the size of the spheroids, can affect the types and levels of trophic factors they secrete (Huang et al., 2019). Furthermore, the pathophysiological environment affects the trophic factors secreted by MSCs. When transplanted into a healthy retina, MSCs release lower levels of BDNF, VEGF, and CNTF compared to the levels released in the retina after optic nerve injury (Millán-Rivero et al., 2018). Alpha RGCs are considered the most resilient to injury following ONC and exhibit the strongest regenerative capacity (Li and Luo, 2024). MSC rescue therapy preferentially protects large-sized RGCs, which are believed to be α-RGCs (Mesentier-Louro et al., 2019; da Silva-Junior et al., 2021). While most studies have observed effects over periods of about a month or shorter, further investigation is necessary through studies with longer observation times. Additionally, understanding the biological mechanisms and signaling pathways underlying the effects of MSCs remains a critical focus of ongoing research.

### Neural stem/progenitor cells

Initially, neural progenitor cells were thought to be derived from neural stem cells (NSCs) and to possess a more limited capacity for self-renewal compared to NSCs. However, these two terms are now often used interchangeably. In this review, we combine them into a single term: NSPCs. NSPCs play a crucial role in generating new neural cells in response to injury or diseases that cause neuronal loss and neuroinflammation in the adult central nervous system (Nemati et al., 2022). They have the capacity to self-renew, proliferate, and differentiate into both neurons and glial cells (Feng et al., 2021). NSPCs are primarily found in two specific areas of the adult brain: the subventricular zone of the lateral ventricles and the subgranular zone of the hippocampus. Additionally, they can be derived from pluripotent stem cells, such as ESCs, iPSCs, and MSCs (Ng et al., 2019; Kim et al., 2021a; Soto et al., 2021). It takes approximately 10 days for hESCs to differentiate into NSPCs, which typically form rosette-like structures. The hESC-derived NSPCs express neural progenitor markers such as paired box gene 6 (PAX6), SRY-box transcription factor 1 (SOX1), SRY-box transcription factor 2 (SOX2), and neuroepithelial stem cell intermediate filament (NESTIN) at both genetic and protein levels (Do et al., 2021; Park et al., 2021; Nemati et al., 2022). These NSPCs can spontaneously differentiate into cells expressing neuronal markers such as TUJ1 and MAP2, confirming their potential to differentiate into neurons (Do et al., 2021). Studies have shown that hESC-derived NSPCs can contribute to the treatment of TON through two mechanisms: secreting neurotrophic factors and regulating the expression of inflammatory factors as a rescue therapy, and integrating into the visual pathway to connect the injury site to the visual center (**Table 3**). In the ONC model, hESC-derived NSPCs can protect RGC survival and increase the expression of GAP43, facilitating axonal extension and regeneration (Park et al., 2021; Shin et al., 2023). The mechanisms underlying these neuroprotective and regenerative effects include enhancing neuroprotective factors such as BDNF, maintaining mitochondrial homeostasis, and activating Wnt/β-catenin signaling (Shin et al., 2023). Furthermore, hESC-derived NSPCs appear to exert more effective neuroprotective and regenerative effects compared to PMSCs (Park et al., 2021). Nemati et al. (2022) administered intravenously transplanted hESC-derived NSPCs into ONC mice and found that these cells provided a neuroprotective effect on the injured retina by promoting RGC and axonal survival. However, this intervention did not result in significant improvements in visual function, as assessed by the visual cliff test (Nemati et al., 2022). In addition to improving RGC survival and axonal regeneration, hESC-derived NSPCs have been found to act as a “neuronal relay.” Following transplantation, they can differentiate into neurons, extend axons that reach the optic chiasm within 4 weeks, facilitate RGC axon extension into the injured area, and receive synaptic inputs from regenerating RGC axons in the optic nerve transection model (Do et al., 2021). Embryo-derived retinal stem cells exhibit characteristics similar to hESC-derived NSPCs, including stem cell-like morphology and high expression of Pax6 and Nestin. Following intravitreal injection into ONC mice, these cells migrate into the host GCL and inner nuclear layer, leading to a significant increase in a-wave amplitudes in electroretinography compared to the PBS-treated group, indicating their potential for repairing the injured retina (Feng et al., 2021). Our previous study demonstrated that niobium carbide MXene promotes the differentiation of RPCs by activating the phosphatidylinositol 3-kinase/protein kinase B (PI3K/Akt) and MAPK/ERK pathways, enhancing RPC migration ability (Tang et al., 2023). Additionally, it scavenges free radicals generated by oxidative stress and improves the cellular microenvironment, providing a more favorable condition for RPCs. This may have similar effects in NSPC therapy, although further studies are needed to confirm this.

**Table 3:** NSPC transplantation for TON treatment

Cell type	Species	Administration	Model	Dosage/eye	Integration	Observation period	Molecular mechanism	Conclusion	Reference
ESC-derived NSPC	Human	Subtenon injection	ONC	2 × 10^6^ cells	NA	4 wk	Enhancing neuroprotective factors and regulating inflammation	Neuronal regeneration–related gene recovery was pronounced in the ONC model post injection.	Shin et al., 2023
ESC-derived NSPC	Human	Intravenous injection	ONC	5 × 10^4^ cells	NA	60 d	NA	RGC protection in the hESC-dervied NSPC group was over twice that of the vehicle group, as indicated by BRN3A staining and retrograde tracing.	Nemati et al., 2022
ESC-derived NSPC	Human	Subtenon injection	ONC	2 × 10^6^ cells	NA	4 wk	hESC-derived NSPCs-induced the expression of neuronal regeneration markers GAP43, THY1, and neurofilaments	hESC-derived NSPCs demonstrated beneficial effects on ONC animal model, showing neuroprotective and pro-regenerative effects.	Park et al., 2021
ESC-derived NSPC	Human	The optic nerve at the crush site	Optic nerve transection	2.5 × 10^6^ cells	Yes	4 wk	NA	hESC-derived NSPC grafts modestly improved RGC survival and enhanced axonal regeneration following optic nerve transection.	Do et al., 2021
RSCs	Mice	Intravitreal injection	ONC	6 × 10^4^ cells	Yes	2 wk	NA	Embryonic RSCs could potentially repair damaged retinas when transplanted via intravitreal injection.	Feng et al., 2021

Different cell types used in NSPC therapy, along with details regarding their administration, models, dosages, and observation periods, are summarized. The effects of NSPC therapy, including potential integration with the host retina, as well as the underlying mechanisms, are also provided. BRN3A: Brain-specific homeobox/POU domain protein 3a; ESC: embryonic stem cell; GAP43: growth associated protein 43; hESC: human embryonic stem cell; NA: not applicable; NSPC: neural stem/progenitor cell; ONC: optic nerve crush; RGC: retinal ganglion cell; RSC: retinal stem cell; THY1: Thy-1 cell surface antigen; TON: traumatic optic neuropathy.

Research on the use of NSPCs as a stem cell therapy for TON is still limited, while their application in spinal cord injury is more established (de Freria et al., 2021). Given that the optic nerve and retina are part of the central nervous system, they may share similar characteristics with spinal cord injuries in terms of repair and regeneration. Notably, hESC-derived NSPCs have demonstrated neuroprotective effects in the ONC model, even when administered intravenously. Moreover, they have shown superior effects compared to PMSCs. Their neuroregenerative potential promotes the extension of RGC axons beyond areas of glial activation (Do et al., 2021). Additionally, NSPCs appear to function as a neuronal relay between RGCs and the visual center. However, further studies are needed to explore whether NSPCs derived from other sources may provide improved outcomes and to elucidate the underlying mechanisms of their effects. Electrophysiological and visual function tests are also essential after hESC-derived NSPCs induce structural repair. Recent studies have observed outcomes from 28 to 60 days post-injury (**Table 3**), underscoring the importance of long-term observation in determining whether NSPCs can be a viable and effective stem cell therapy for TON.

### Cell-derived products

While cell therapy holds great potential, it also carries risks such as transplant rejection, small vessel obstruction, and tumor formation (Volarevic et al., 2018). In this context, cell-derived products have been proposed as a promising alternative (**Table 4**). EVs are nanoscale, phospholipid bilayer-enclosed particles that are derived from the inward budding or outward curvature of the plasma membrane. These vesicles carry bioactive factors such as miRNAs, proteins, mRNAs, lipids, carbohydrates, and metabolites (Su et al., 2024; Yi et al., 2024). Based on their size, internal cargo, and surface proteins, EVs can be classified into exosomes, microvesicles, and apoptotic bodies, each with distinct biosynthetic functions. Exosomes range in diameter from approximately 30 nm to 150 nm, while microvesicles and apoptotic bodies range from 100 nm to 1000 nm and from 100 nm to 5000 nm, respectively (Li et al., 2022a). However, the Minimal Information for Studies of Extracellular Vesicles 2018 (MISEV2018) guidelines suggest using operational terms like “small EVs” (sEVs) for particles < 200 nm, based on size, density, or biochemical composition, rather than terms such as exosomes (Théry et al., 2018). EVs are produced by various cells in the body, including stem cells, glial cells, tumor cells, mast cells, and T cells (Koh et al., 2020; Zhu et al., 2023). There is substantial evidence supporting the idea that EVs play a pivotal role in facilitating cell-to-cell communication, coordinating complex interactions, and stabilizing a dynamic, homeostatic microenvironment (Cui et al., 2021; Su et al., 2024). EVs can be efficiently internalized by target cells, where they induce specific cellular effects (Mathew et al., 2021). They play roles in neuroprotection, immunomodulation, anti-inflammation, repair, and regeneration by mediating the paracrine effects of MSCs and other cells (Cui et al., 2021). Furthermore, EVs are capable of transporting therapeutic molecules into cells (Wang et al., 2021).

**Table 4:** Cell-derived product for TON treatment

Origin of products	Species	EV biomarker	Negative biomarker	Administration	Dosage/eye	Study type	Model	Observation period	Molecular mechanism	Conclusion	Reference
hiPSC-NPSC	Human	SDCBP, TSG101 and ALIX	Calnexin	Intravitreal injection	2 × 10^11^ particles	*In vivo*	ONC	14 d	Delivered neuroprotective and anti-inflammatory miRNAs to target cells.	Reduced RGC loss significantly restored visual function partially, caused significantly potent and durable neuroprotective effects.	Li et al., 2025
Immortalized MSC	Mouse	Alix, CD9, and CD81	NA	Intravitreal injection	1 × 10^8^ particles every 3 d for a total of 14 d	*In vivo*	ONC	28 d	Triggering G-CSF release and facilitating Ly6Clow Mo/MΦ recruitment.	Promoted both RGC survival and axon regeneration through immunomodulatory pathway.	Yi et al., 2024
Schwann cell	Rat	CD63, TSG101, and Alix	Calnexin	Intravitreal injection	4.5 μg	*In vitro* and *in vivo*	ONC	14 d	Activated the cAMP-response element binding protein signaling pathway and regulated reactive gliosis.	Mitigated RGC degeneration, prevented RGC loss, and preserved the GCC thickness in a rat ONC model.	Zhu et al., 2023
UC-MSC	Human	CD9, CD63 and TSG101	NA	Intravitreal injection	3 × 10^9^ particles	*In vitro* and *in vivo*	ONC	21 d	Via regulating miR-222-3p and miR-22-3p to promote axon regeneration by activating mTORC1.	Increased RNFL thickness, promoted axon regeneration and recovered RGC function efficiently	Sang et al., 2023
PMSC	Human	CD9, CD81, and CD63	NA	Subtenon injection	300 μg	*In vitro* and *in vivo*	Optic nerve compression	4 weeks	Activated the LONP1/p62 signaling pathway. Significantly increased the levels of the antioxidants peroxiredoxin 2 and peroxiredoxin5.	Attenuated the hypoxic injury of RGC and facilitated recovery of damaged axons in ONC.	Park et al., 2022
BMSC	Rat	CD63 and TSG101	GM130	Intravitreal injection	3 × 10^9^ particles	*In vivo*	ONC	30 d	MSC-exos improved antiapoptotic protein Bcl-2 expression and decreased pro-apoptotic protein Bax expression, and cleaved caspase-3 activation.	Promoted the survival of RGCs in ONC rats notably.	Cui et al., 2021
ESC-MSC	Human	CD81, TSG101, and CD63	Calnexin	Intravenous injection	15 μg on d 2, d 4, and d 6	*In vivo*	ONC	60 d	Cis p-tau, a central mediator of neurodegeneration in injured RGCs, was downregulated.	Improved Brn3a^+^ RGC survival and both retro- and anterograde RGC tracing, prevented RNFL degenerative thinning, and promoted GAP43^+^ axon growth in the optic nerve.	Seyedrazizadeh et al., 2020
UC-MSC	Human	CD81 and CD63	NA	Intravitreal injection	1 × 10^9^ particles on d 0, d 7, and d 14	*In vivo*	ONC	21 d	NA	Promoted Brn3a^+^ RGC survival in the retinal ganglion cell layer, enhanced GFAP^+^ glia cell activation in the retina and optic nerve	Pan et al., 2019
RGC	NA	Alix, CD 63, CD 81, and CD9	Cytochrome C	Intravitreal injection	4 μL of EV- loaded PACAP38 (20 μM)	*In vivo*	ONC	14 d	NA	Enhanced the RGC survival rate and RNFL thickness	Wang et al., 2021
Amnion-derived multipotent progenitor cell	Human	NA	NA	Intranasal Delivery	20 μL of undiluted ST 266 daily for either 5 or 10 d	*In vivo*	ONC	10 d	NA	Preserved visual function and reduces RGC damage and loss of myelin in the optic nerve	Grinblat et al., 2018
Subcutaneous adipose tissue	Human	NA	NA	Intravitreal injection	15 μg of CEFFE	*In vivo*	ONC	17 d	Inhibited microglia activation and elevated anti-inflammatory factors.	Promoted axon re-generation and enhanced RGCs survival	Sun et al., 2024

An overview of various sources of cell-derived products used in cell-based therapy for TON is provided, including relevant details on administration, study types (*in vivo* or *in vitro*), rodent models, dosages, and observation periods. Positive and negative markers of EVs are also included. Additionally, the effects of cell-derived product transplantation and the underlying mechanisms are highlighted. ALIX: Apoptosis-linked gene 2-interacting protein X; Bax: Bcl-2-associated X protein; Bcl-2: B-cell lymphoma 2; BMSC: bone marrow-derived mesenchymal stem cell; Brn3a: brain-specific homeobox/POU domain protein 3a; cAMP: cyclic adenosine monophosphate; CEFFE: cell-free fat extract; ESC-MSC: embryonic stem cell-derived mesenchymal stem cell; EV: extracellular vesicles; GAP43: growth-associated protein 43; GCC: ganglion cell complex; G-CSF: colony-stimulating factor 3; GFAP: glial fibrillary acidic protein; hiPSC-NPSC: human induced pluripotent stem cell-derived neural stem/progenitor cell; LONP1: lon peptidase 1; MSC: mesenchymal stem cell; mTORC1: mechanistic target of rapamycin complex 1; NA: not applicable; ONC: optic nerve crush; PACAP38: pituitary adenylate cyclase-activating polypeptide-38; PMSC: placenta-derived mesenchymal stem cell; RGC: retinal ganglion cell; RNFL: retinal nerve fiber layer; SDCBP: syndecan binding protein 1; TON: traumatic optic neuropathy; TSG101: tumor susceptibility gene 101; UC-MSC: umbilical cord blood-derived mesenchymal stem cell.

Compared with MSCs, EV therapy offers several advantages:

1. Safety: EVs cannot replicate, alter their phenotype, or actively migrate, thereby avoiding the risks of tumor and ectopic tissue formation. Since they do not divide, the dosage of EV injections can be precisely controlled (Park et al., 2022).

2. Specificity: EVs have a migration advantage, enabling them to cross the vitreous and enter target tissues more effectively than parent MSCs (Cui et al., 2021; Mathew et al., 2021). Moreover, EVs can be engineered with specific proteins to enhance their targeting specificity (Sen et al., 2023).

3. Low immunogenicity: EVs are relatively less immunogenic, reducing the risk of transplant rejection (Mathew et al., 2021; Li et al., 2025).

4. Ease of isolation and storage: EVs can be easily isolated through ultracentrifugation, the most commonly used method, and stored with minimal effort (Seyedrazizadeh et al., 2020; Tieu et al., 2020). They can also be sterilized through filtration due to their small size (Park et al., 2022).

These advantages position EVs as a promising therapeutic option for clinical applications.

EV-based therapy has also shown benefits in the TON model. In the ONC model, the injection of small EVs derived from hiPSC-NSPCs (hiPSC-NSPC-sEVs) can increase the mean thickness of the GCC layer, promote the survival of RGCs, and partially restore visual function, suggesting a neuroprotective effect of hiPSC-NSPC-sEVs. This effect is reportedly mediated by the inhibition of excessive microglial activation through the transport of neuroprotective and anti-inflammatory miRNAs. Additionally, since hiPSC-NSPCs can differentiate into neuronal cells, the neuroprotective effect of hiPSC-NSPC-sEVs is more potent and durable compared to hiPSC-sEVs (Li et al., 2025). MSC-derived sEVs (MSC-sEVs) exhibit similar neuroprotective effects, promoting RGC survival and axon regeneration in the ONC model. MSC-sEVs are primarily taken up by retinal vascular mural cells, which stimulate the release of colony-stimulating factor 3, recruiting a restorative Ly6C^low^ Mo/MΦ population to repair the optic nerve injury site (Yi et al., 2024). BMSC-derived sEVs also promote RGC survival through anti-inflammatory and anti-apoptotic effects. Intravitreal injection of BMSC-derived sEVs has been shown to decrease pro-inflammatory cytokine levels while increasing anti-inflammatory factors. Additionally, BMSC-sEVs stimulate protein kinase B phosphorylation, increase the Bcl-2/Bax ratio, and downregulate caspase-3 activity, mitigating apoptosis induced by ONC (Cui et al., 2021). A previous study found that human umbilical cord mesenchymal stem cell-derived sEVs (hUC-MSC-sEVs) protect RGC survival but do not improve axon regeneration. This study also observed increased glial activity in both the retina and optic nerve *in vivo* in the hUC-MSC-sEV-treated group. More recently, Sang et al. (2023) demonstrated that hUC-MSC-sEVs not only promote RGC survival and axon regeneration but also increase the thickness of the RNFL and restore RGC function, as monitored by pattern electroretinography, 21 days after ONC. These effects are potentially mediated by miR-222-3p and miR-22-3p through the activation of the mechanistic target of rapamycin complex 1 (mTORC1) pathway. When interpreting the discrepancies between these two studies, several factors should be considered. In Sang’s study, a single dose of 3 × 10^9^ EVs was administered, whereas in the previous study, 1 × 10^9^ EVs were injected on days 0, 7, and 14 after ONC. This suggests that a single, higher dose may be more effective than multiple lower doses. Furthermore, the severity of ONC affects treatment efficacy (Grinblat et al., 2018). The duration and intensity of pressure applied to the optic nerve in the ONC model used in the earlier study may have been greater than in Sang’s study, potentially contributing to the observed differences in outcomes. Glial fibrillary acidic protein (GFAP)–positive astrocytes play dual roles in axon regeneration, exhibiting both supportive and inhibitory functions (Anderson et al., 2016). The increased glial activity observed in the earlier study may have contributed to a more inhibitory environment, thereby impeding regeneration.

After hypoxic preconditioning, hPMSC-derived sEVs mitigate hypoxic injury in R28 RPCs by upregulating the lon peptidase 1/p62 signaling pathway. *In vivo*, these sEVs increase the number of RGCs in an ONC model, an effect not observed without hypoxic preconditioning (Park et al., 2022). Therefore, hypoxic preconditioning is recommended when utilizing pluripotent stem cells and their derivatives. HESC-derived MSCs also secrete EVs that enhance RGC survival, promote axon neurogenesis in RGCs, and inhibit RNFL thinning due to degeneration, with effects observed 60 days after intravenous injection in an ONC rodent model. Additionally, hESC-MSC-EVs can significantly improve cognitive visual behavior, potentially by decreasing cis p-tau and tauopathy in RGCs (Seyedrazizadeh et al., 2020). In addition to stem cell-derived sEVs, Schwann cell-derived sEVs (SC-sEVs) are internalized by primary RGCs within 48 hours, promoting their survival and axon growth *in vitro*. In a rat ONC model, SC-sEVs are taken up within 24 hours, leading to reduced RGC degeneration and loss, preservation of GCC thickness, and enhanced axon regeneration even 14 days after injection. The cyclic AMP response element-binding protein signaling pathway plays a crucial role in SC-sEV-mediated neuroprotection by preventing RGC apoptosis and increasing reactive gliosis (Zhu et al., 2023).

Overall, sEVs derived from various cell types appear to benefit RGC survival and axon regeneration in ONC models. Additionally, sEVs can serve as effective delivery vehicles in TON models. For instance, sEVs loaded with PACAP38 via CP05 (an exosomal anchor peptide that facilitates binding to both the exosomal surface protein CD63 and PACAP38) exhibit an 87.1% binding efficiency and are taken up by 86.9% of RGCs *in vitro*. Moreover, sEVs loaded with PACAP38 increase RGC survival, enhance RNFL thickness, and promote axon regeneration and optic nerve function when intraocularly injected into a rat ONC model. The use of sEVs as carriers addresses the challenges of low tissue penetration and short half-life of these molecules (Wang et al., 2021).

In addition to EVs, solutions extracted from pluripotent stem cells can also exhibit neuroprotective effects. The cell-free fat extract (CEFFE), derived from human subcutaneous adipose tissue, contains a rich array of inflammatory factors, neurotrophic factors, and chemokines. It has been reported that CEFFE promotes RGC survival and axon regeneration when administered three times following ONC injury, demonstrating superior effects compared to single neurotrophic factor treatments (Sun et al., 2024). CEFFE also exhibits anti-inflammatory properties, reduces microglial activation, enhances mammalian target of rapamycin expression, and lowers Rho associated coiled-coil containing protein kinase 2 expression, thereby promoting axon regeneration in the injured tissue. ST266, another cell-free amnion-derived cellular factor solution, has the capacity to mitigate RGC damage, reduce demyelination, and preserve visual function after intranasal administration in an ONC model *in vivo* (Grinblat et al., 2018).

On the whole, EVs derived from hiPSC-NPSCs, immortalized MSCs, BMSCs, hUC-MSCs, hPMSCs, ESC-MSCs, and Schwann cells are utilized in preclinical experiments for the treatment of TON. Representative biomarkers such as apoptosis-linked gene 2-interacting protein X (ALIX), tumor susceptibility gene 101 (TSG101), CD9, CD81, CD63, and syndecan binding protein 1 (SDCBP), along with negative biomarkers such as calnexin, golgi matrix protein 130 (GM130), and cytochrome C, are used to confirm that the isolated particles are indeed EVs and do not contain organelles. The injection routes include intravitreal, intravenous, subtenon, and intranasal delivery, with intravitreal injection being the most commonly used method (**Table 4**). Following ONC, degeneration is progressive and difficult to halt; therefore, most studies administer cell-derived products immediately after ONC to maximize therapeutic effects (Cui et al., 2021; Sun et al., 2024; Li et al., 2025). Two studies administer treatment three times at specific intervals (Pan et al., 2019; Seyedrazizadeh et al., 2020). After injection, these EVs have been shown to cross the inner limiting membrane (ILM) and be ingested by retinal mural cells, as well as various retinal cells, including RGCs, microglia, astrocytes, and Müller cells, particularly those in the GCL, inner plexiform layer, and inner nuclear layer (Mathew et al., 2021). EVs appear to be internalized by the first cell they encounter, exhibiting no selectivity, in a dose-dependent and capacity-limited manner (Pan et al., 2019; Mathew et al., 2021; Zhu et al., 2023). However, the duration of retention varies among different retinal cell types, with the longest persistence observed in RGCs, lasting up to 14 days (Mathew et al., 2021; Li et al., 2025). The therapeutic effect is dose-dependent, but there is a limit—more does not necessarily equate to better outcomes (Sang et al., 2023). The effects of EVs *in vitro* appear to be more pronounced than *in vivo*, as additional factors in the *in vivo* environment may influence the uptake process (Sang et al., 2023; Zhu et al., 2023). The dose-response relationship in cell-derived product therapy remains unclear, and further studies are needed to determine the optimal timing and dosage for treatment. Since EVs are rapidly cleared from the vitreous and cannot serve as a long-term reservoir, it is also important to explore ways to improve the delivery and retention of EVs (Mathew et al., 2021). Biomaterials may offer a potential solution (Lu et al., 2022). EVs from different sources exhibit varying effects; for example, hiPSC-NPSC-derived sEVs are more effective in neuroprotection than hiPSC-derived EVs, and hUC-MSC-derived EVs outperform BMSC-derived EVs in axon regeneration (Sang et al., 2023; Li et al., 2025). The retinal microenvironment can influence the therapeutic efficacy of cell-derived products. EVs derived from different sources have been shown to reduce microglial activation, stimulate astrocytes and Müller cells to release neurotrophic factors, or promote beneficial neuroinflammation, thereby contributing to neuroprotection and neurogenesis (Zhu et al., 2023; Yi et al., 2024; Li et al., 2025). Therefore, it is crucial to clarify how the roles of Müller glial cells, astrocytes, and microglia, as well as the processes of glial activation and neuroinflammation, affect neuroprotection and neuroregeneration. Hypoxic and TNF-α preconditioning have been reported to enhance the neuroprotective effects of EVs produced under these conditions (Mead et al., 2020; Park et al., 2022). In the current literature, the longest observation period for cell-derived therapies in the treatment of TON is 60 days, while most studies observe for less than 30 days (**Table 4**). Extended observation periods are necessary to fully assess the safety and efficacy of this therapeutic approach.

## Clinical Trials

Stem cell therapy is a promising approach for treating TON. Among the studies registered in ClinicalTrials.gov and ChiCTR.org.cn (accessed on January 21, 2025), two studies are related to TON (**Table 5**).

**Table 5:** Registered clinical trials of stem cell therapy for TON

Identifier	Performers	Current Status	Intervention	Study phase	Study type	Number of patients	Study start date	Estimated trial end date	Results	Reference
ChiCTR-TRC-14005093	Research Institute of Surgery, Daping Hospital, Third Military Medical University, Chongqing	Completed	Local UC-MSC transplantation	Phase I	Open-label, single-center, prospective	20	September 1, 2014	July 1, 2016	No any systemic or ocular complications. No adverse events related to local transplantation. No significant change in best-corrected visual acuity at follow-up with or without local UC-MSC transplantation.	Li et al., 2021
20150196587 (Korean FDA), 2015-07-123-054 (IRB), NCT05147701	Department of Ophthalmology, CHA Bundang Medical Center, CHA University, Seongnam-si, Gyeonggi-do 463 712, Republic of Korea	Recruiting	Subtenon transplantation of hPMSCs	Phase I	Open label, single center, nonrandomized clinical trial	5	NA	NA	No evidence of adverse proliferation, tumorigenicity, severe inflammation or other serious issues during the 12-mon follow-up period. Visual acuity improved in all four patients.	Sung et al., 2020

Clinical trials on cell-based therapy for TON registered on ClinicalTrials.gov and ChiCTR.org.cn are listed in this table. The details, including the trial identifier, researchers, current status, intervention, study phase, study type, number of participants, study start date, estimated trial end date, and results, are summarized. hPMSC: Human placenta-derived mesenchymal stem cell; NA: not applicable; UC-MSC: umbilical cord-derived mesenchymal stem cell.

A Phase I study (ChiCTR-TRC-14005093) conducted in China utilized gelatin sponge as a scaffold for MSCs. The MSC-gelatin sponge scaffold was transplanted onto the injured site after optic nerve decompression in 10 patients with TON. Over the following 6 months, no adverse events associated with MSC transplantation were observed. Recovery in the MSC transplantation group lasted for 1 month, while recovery in the control group lasted only 1 week, potentially due to the neuroprotective effects of MSCs. However, there was no significant difference in vision improvement between patients who received MSC transplantation along with optic nerve decompression and those who underwent only optic nerve decompression. This lack of significant difference may be attributed to the small sample size and the poor initial vision of the patients before the intervention (Li et al., 2021).

Another Phase I study (NCT05147701; 20150196587, Korean FDA) enrolled five patients with TON, although one patient dropped out due to concomitant foveal ellipsoid zone disruption in the affected eye. Subtenon transplantation of hPMSCs was performed on the remaining four patients. No adverse proliferation, tumor formation, severe inflammation, or other serious complications were observed. Visual acuity improved over the 12 months following transplantation (Sung et al., 2020).

## Challenges and Prospectives

### Cell sources

The primary sources of stem cell therapy for TON include ESCs, iPSCs-derived RGCs, MSCs, and NSPCs. Among these, ESCs/iPSCs-derived RGCs are considered capable of repairing the injured retina both morphologically and functionally. Historically, the efficiency of differentiating ESCs/iPSCs into RGCs has been regarded as low (Hua et al., 2020). However, recent studies indicate that ESCs/iPSCs can be converted into RGC-like cells with an efficiency exceeding 89.5% within 10–20 days (Luo et al., 2022; Liou et al., 2023). Furthermore, the efficiency of chemical induction differentiation has also reached between 82% and 87% (Vrathasha et al., 2022). Biomaterial scaffolds that promote stem cell differentiation may also help shorten the differentiation time (Chen et al., 2019b). The purity of donor RGC-like cells is crucial to mitigate the risk of teratoma formation caused by residual stem cells. The methods for purifying RGCs have been refined, including MACS using THY1 and FACS with markers such as CD184 and CD171 for the selection of RGC-like cells (Rabesandratana et al., 2020; Li and Luo, 2024). However, a separate study reported tissue overgrowth when using MACS with THY1 as the isolation marker (Oswald et al., 2021). Further research is needed to explore additional markers to achieve higher purification efficiency. These advancements may facilitate the translational pipeline for cell replacement therapy in TON. ESCs present challenges in obtaining specific cell types and raise ethical concerns, while iPSCs may introduce genomic instability, increasing the risk of tumor formation. Additionally, the roles of microglia and macrophages in functional modulation should be considered in treatments for TON. Notably, adult mouse Müller glia can be reprogrammed into functional RGC-like cells through the use of NGN2 (Guimaraes et al., 2018). Further research is required to refine the therapeutic effects of these reprogrammed RGC-like cells for treating TON. In addition to stem cells, cell-derived products such as EVs and extracts have also demonstrated neuroprotective effects in TON models. Future studies should investigate which components of EVs and which specific ingredients in extracts contribute to these therapeutic effects.

### Transplantation procedure

The dosage and route of administration must be carefully considered for stem cell and cell-derived product transplantation. The quality and condition of stem cells derived from various sources can vary significantly, leading to differences in reported dosages for transplantation (**Tables 1–3**). However, evidence suggests that higher dosages of stem cells are associated with prolonged residence time at the injection site and enhanced therapeutic effects (Mesentier-Louro et al., 2019; da Silva-Junior et al., 2021). Furthermore, different delivery methods may influence the optimal dosage of cells in cell therapy (Looi et al., 2022). Overall, the dosage for intravenous injection tends to be higher than for subtenon injection, while the latter is higher than for intravitreal injection (**Table 2**). It is crucial to determine an appropriate dosage for cell transplantation in preclinical studies. Future research may also involve repeated stem cell injections with extended follow-up periods to establish the optimal dosage required for functional efficacy.

In addition to dosage, the choice of cell delivery routes can significantly affect the safety and efficacy of cell therapy. The delivery routes utilized in stem cell therapy include intravitreal, subtenon, intravenous, peribulbar, retrobulbar, endonasal, and intrathecal approaches (Labrador-Velandia et al., 2016), with the first four being the most commonly used in treating TON (**Tables 1–4**). Intravitreal injection is the most targeted approach for delivering cells to the retina, avoiding disruption of the retinal barrier and minimizing systemic exposure (Varela-Fernández et al., 2020). Subtenon injection can also deliver cells to the posterior segment of the eye and is considered less invasive and safer for repeated treatments compared to intravitreal and intravenous injections (Park et al., 2021). For delivering cell-derived products, intranasal administration may be used, allowing for more frequent treatments while reducing discomfort and minimizing the risk of complications (Grinblat et al., 2018). Our previous study demonstrated that injectable hydrogels composed of chitosan hydrochloride and oxidized dextran biomaterials can mitigate injury to transplanted RPCs during the injection process, providing valuable insights for further research on cell-based therapy for TON (Jiang et al., 2019). Further studies should explore the optimal administration route along with the corresponding dosage to establish a standardized treatment option for TON, especially when transplantation involves biomaterials.

### Integration of transplanted cells

To achieve sustained therapeutic effects in retinal transplantation, it is crucial to prolong cell survival at the transplantation site. Recent advances in single-cell RNA sequencing have identified a host of genes and molecules that may enhance the survival of RGCs and counteract the inhibitory effects of glial scars and myelin-associated factors (Li et al., 2022b). Current studies have varied observation periods post-transplantation, ranging from 7 to 240 days, with the majority focusing on effects observed around 30 days (Mesentier-Louro et al., 2019; Zhang et al., 2020; Mathew et al., 2021; **Tables 1–4**). Further long-term experiments are essential not only to assess the duration of neuroprotection provided by MSCs, NSPCs, and cell-derived products but also to evaluate axon growth, synapse formation, and the functionality of stem cell-derived RGCs.

The migration, integration, and connection of transplanted cells with the host retina remain significant challenges in stem cell therapy. The ILM acts as a physical barrier, hindering the migration and integration of both stem cells and stem cell-derived RGCs when transplanted into the vitreous (Zhang et al., 2021c). Disruption of the ILM has been shown to facilitate the migration of intravitreally injected cells (Li and Luo, 2024). The ILM is a basement membrane composed of ECM proteins, including collagen IV, laminins, nidogens, and heparan sulfate proteoglycans, with its retinal surface formed by the plasma membrane of Müller glial footplates (Zhang and Johnson, 2021). Johnson et al. (2010) demonstrated that the migration of grafted cells was enhanced when the ILM was peeled or when Müller cell function was disrupted by α-aminoadipic acid, but not when the basal lamina was degraded by collagenase. They suggested that glial reactivity, rather than the ECM of the inner basal lamina, played a pivotal role in limiting grafted cell migration. Zhang et al. (2021c) reported that pretreatment with pronase E, which effectively disrupts the ILM while preserving glial reactivity, can enhance the ingrowth of retinal neurites from exogenous RGCs in *ex vivo* organotypic retinal explants. Pre-injecting or co-injecting agents such as pronase E and α-aminoadipic acid may facilitate the integration and connection of stem cells with the host retina (Johnson et al., 2010; Zhang et al., 2021c). Nevertheless, it is crucial to note the potential adverse effects *in vivo* that may arise from excessive enzyme use, including cataract formation, inflammation, and both vitreal and subretinal hemorrhaging (Johnson et al., 2010; Vrathasha et al., 2022). Additionally, an intact ILM seems to promote the clustering of RGC-like cells, although the exact significance of this remains unclear (Zhang et al., 2021b, c). In summary, while the ILM is considered a barrier to the integration of transplanted RGCs, gaining insight into RGC-ILM interactions and their involvement in neuroretinal development is essential. The main challenge is finding methods to facilitate the passage of grafted cells through the ILM and their integration into the host retina without fully compromising the ILM.

The engrafted cells are initially injected into the vitreous without physical or trophic support before attaching to and integrating with the retina (Wu et al., 2018). Biomaterials, such as scaffolds, can play a critical role in supporting the survival, proliferation, and differentiation of these cells (Chen et al., 2019b). Furthermore, transplanting cells in a scaffold designed as a cell sheet structure may enhance integration and positioning within the host retina (Luo et al., 2021). A recent study demonstrated that polydopamine nanocomposite scaffolds can regulate the retinal microenvironment, facilitate the polarization of retinal microglia/macrophages toward the M2 phenotype, and promote RGC survival and axon regeneration (Pan et al., 2024).

Following the transplantation of stem cell-derived RGCs, axon regeneration is a crucial step toward enhancing vision; however, it is a complex process influenced by various intrinsic and extrinsic factors. Notably, the capacity for axon regeneration differs among the various subtypes of RGCs (Yuan et al., 2021). Many regenerated axons do not navigate properly between the optic nerve and the brain, necessitating the development of methods to guide axon growth. Electrical fields and scaffolds can play a pivotal role in this process. RGC axons demonstrate growth directed toward the cathode when exposed to an electric field *in vitro* (Gokoffski et al., 2019). The aligned poly(glycerol sebacate)/poly(ε-caprolactone) scaffold has been reported to provide a suitable structure for RGC attachment and growth, promoting axon extensions and neurite outgrowth (Behtaj et al., 2021). The 3D-structured PBG scaffold containing glutamate has also been shown to enhance and guide the neurite outgrowth of hiPSC-derived RGC progenitors, serving as both a physical and chemical cue for neurite alignment and extension (Chen et al., 2019b). Additionally, scaffolds with customized micro- and nano-grooving created using 3D printing technology can enhance axon directionality and function (Yang et al., 2017). Light stimulation and pulsed magnetic field stimulation may also aid in synapse formation (Gokoffski et al., 2020). When regenerated RGC axons connect with their corresponding brain areas and establish signal transmission, this leads to a partial restoration of visual function. However, the axons of regenerated RGCs produced by current techniques for enhancing optic nerve regeneration largely remain unmyelinated, which limits their effectiveness in supporting visual functions. Further studies are needed to explore methods for regenerating axons with a myelin sheath and to determine the extent of axon regeneration required for meaningful improvement in vision.

Stem cell therapy cannot eliminate the risks of inflammation following cell administration, and neuroprotective agents may be beneficial. Additionally, the success of transplantation and the survival of stem cells can be affected by transplant rejection. Future research should investigate whether immunosuppressive drugs are necessary. Studies have shown that hypoxic preconditioning can enhance the neuroprotective effects of MSCs and EVs (Kwon et al., 2020; Park et al., 2022). Further exploration of the effects of other preconditioning strategies on the neuroprotective properties of stem cells and the underlying mechanisms involved is warranted.

### Clinical translation

Currently, the majority of preclinical research on stem cell therapy for TON relies on cultured cells *in vitro* and small rodents *in vivo*. Before translating findings from rodent studies to humans, it is advisable to conduct tests in primates, which share greater physiological similarities with humans. As previously mentioned, only two clinical trials have been registered on ClinicalTrials.gov and ChiCTR.org.cn (accessed on January 21, 2025). The objectives of Phase I and II clinical trials are to evaluate safety and efficacy. Additional clinical trials are necessary before stem cell therapy can be implemented in clinical practice. ESCs are a valuable source for replacement therapy; however, their widespread clinical use raises significant ethical concerns. In contrast, iPSCs can mitigate these ethical issues and reduce the risk of transplant rejection when derived autogenously, making them a more promising source for stem cell therapy. Nonetheless, the costs associated with generating autogenous iPSCs for each patient are a significant challenge to clinical application. Fortunately, MSCs from various tissues, especially those from adipose tissue, which are more readily accessible, have demonstrated therapeutic effects (Li et al., 2018; Wang et al., 2018). In particular, WJ-MSCs from different umbilical cords have shown similar neuroprotective effects (Millán-Rivero et al., 2018). These findings support the potential for the clinical translation of cell-based therapies.

## Limitations

One limitation of this review is that it only includes articles written in English, focusing on cell-based therapies in models of TON, wild-type models, or clinical trials. The review primarily considers studies on cell-based therapy for TON published between 2018 and January 2025, excluding articles published after this period. As a result, recent developments or studies beyond this timeframe may not be represented. Furthermore, this review specifically focuses on cell-based therapies for TON, covering a total of 11 articles on RGC replacement therapy, 13 on MSC therapy, 5 on NSPC therapy, and 11 on cell-based product therapy. Consequently, studies outside this scope or those exploring other therapeutic strategies may not be included, potentially overlooking significant advancements or alternative approaches. Another limitation is the limited number of articles on NSPC therapy, which may result in an incomplete evidence base.

## Conclusions

**Figure 3:**
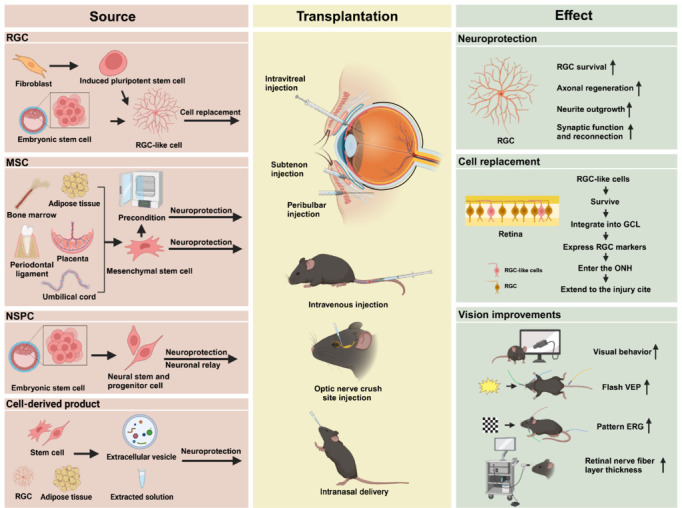
Research progress of cell-based therapies in TON. ESCs and iPSCs can be induced or reprogrammed to differentiate into neurons that resemble RGCs for cell replacement therapy. MSCs, which can be isolated from various tissues, including bone marrow, periodontal ligament, adipose tissue, placenta, and umbilical cord, can also be preconditioned (e.g., under hypoxic conditions) to enhance their neuroprotective effects before transplantation. NSPCs, derived from human ESCs, can provide neuroprotective effects and serve as neuronal replacements. Additionally, cell-derived products, including EVs and solutions extracted from stem cells, RGCs, and other cell types or tissues, can contribute to neuroprotection. When RGC-like cells, stem cells, and cell-derived products are transplanted into models of TON, six commonly used delivery routes are available: intravitreal, subtenon, peribulbar, intravenous, optic nerve crush site injections, and intranasal delivery. Among these, intravitreal injection is the most commonly used route in preclinical research on cell-based therapies for TON. Following the application of cell-based therapy in TON models, improvements are observed in RGC survival, axonal regeneration, neurite outgrowth, synaptic function, and reconnection. RGC-like cells can also survive and integrate into the ganglion cell layer, functioning properly. Additionally, vision improvements have been demonstrated in rodent models. Created with BioRender.com. ERG: Electroretinogram; ESCs: embryonic stem cells; EVs: extracellular vesicles; GCL: ganglion cell layer; iPSC: induced pluripotent stem cell; MSC: mesenchymal stem cell; NSPC: neural stem/progenitor cell; ONH: optic nerve head; RGC: retinal ganglion cell; TON: traumatic optic neuropathy; VEP: visual evoked potential.

TON remains a significant clinical challenge, as there is currently no widely accepted effective treatment available. Additionally, RGC loss and axonal degeneration are progressive and difficult to arrest, while RGCs have a limited capacity for spontaneous regeneration following injury. Over the past few decades, tremendous progress has been made in exploring the potential of cell-based therapies for restoring RGC function and promoting axonal regeneration (**Figure 3**).

Research efforts have significantly advanced our understanding of the mechanisms regulating RGC survival, neuroprotection, and axon regeneration in TON models. Studies have demonstrated that iPSCs and ESCs hold substantial therapeutic promise due to their ability to differentiate into RGC-like cells. RGC-like cells derived from ESCs and iPSCs typically undergo a two-stage differentiation process, in which RPCs mature into RGCs. Recent advancements in using small molecules and peptide modulators to promote RGC differentiation have improved efficiency. Additionally, 3D retinal organoids are being used to better replicate the natural retinal architecture and support RGC development. Direct cellular reprogramming using transcription factors such as NGN2 has also shown high efficiency in generating RGC-like cells. The transplantation of stem cell-derived RGCs has been tested in various animal models, including the ONC rat and wild-type mouse models. Studies have shown that transplanted RGC-like cells can survive and integrate into the host retina, particularly in the GCL, for periods ranging from 3 weeks to 5 months. However, several challenges remain, including the optimization of differentiation protocols, limited survival and integration of transplanted cells, insufficient axonal outgrowth, and functional reconnection with downstream visual pathways. Furthermore, the timing of transplantation and the selection of specific RGC subtypes are likely critical factors in maximizing therapeutic efficacy. Future research should prioritize refining transplantation strategies, elucidating inflammatory responses, and exploring co-transplantation approaches with supportive cells to enhance the survival and integration of transplanted RGCs in optic nerve injury models. MSCs have shown potential in treating TON by promoting RGC survival, axon regeneration, and partial functional recovery. The therapeutic effects of MSCs are attributed to factors such as increased secretion of neurotrophic factors, including BDNF, VEGF, and CNTF, as well as the regulation of inflammatory responses. Additionally, combining MSC therapy with other cell types, such as Schwann cells, or integrating gene therapy has shown promising results. However, challenges remain in optimizing MSC therapy. The survival duration of MSCs can vary significantly; some studies report effects lasting only a few weeks, while others have observed lasting benefits for up to 240 days. Long-term efficacy is influenced by factors such as MSC dosage and delivery routes. Furthermore, the xenogeneic nature of human MSCs may affect their survival in animal models. Inflammatory responses induced by MSCs, such as the activation of Iba1^+^ microglial cells, can disrupt retinal structures and complicate their therapeutic application. Additionally, the optimal culture conditions for MSCs (2D *versus* 3D, hypoxic preconditioning) and their influence on trophic factor secretion require further investigation. Lastly, the biological mechanisms underlying the effects of MSCs are not fully understood, necessitating more in-depth studies to clarify their signaling pathways and improve therapeutic outcomes. NSPCs play a vital role in repairing the central nervous system by differentiating into neurons and glial cells. Derived from sources such as hESCs, NSPCs promote RGC survival and axonal regeneration in TON models. Studies have demonstrated their neuroprotective effects, including the enhancement of neurotrophic factors and the activation of signaling pathways such as Wnt/β-catenin. NSPCs also function as a “neuronal relay,” connecting injured retinal areas to the visual pathway. However, there is a limited amount of research on NSPC therapy for TON. Although hESC-derived NSPCs show promise, their long-term efficacy in improving visual function remains unclear, and further studies are needed to assess the potential of NSPCs from different sources. Comprehensive electrophysiological and visual function tests are essential to evaluate the therapeutic outcomes of NSPCs, particularly with longer observation periods, to determine their viability as a treatment for TON. Cell-derived products, particularly EVs, are emerging as a promising alternative to cell-based therapies for treating TON. EV therapy has demonstrated neuroprotective effects in TON models by promoting RGC survival and axon regeneration through mechanisms such as inhibiting microglial activation, reducing inflammation, and transporting therapeutic molecules. Studies have shown the efficacy of small EVs derived from various sources, including hiPSC-NSPCs, MSCs, and Schwann cells, in improving RGC survival and function. However, several challenges hinder the widespread clinical application of EV-based therapy. Despite their potential, the dose-response relationship in EV therapy remains unclear, and therapeutic effects observed *in vitro* often do not translate directly to *in vivo* settings due to additional factors influencing EV uptake and retention in living organisms. While EVs from various sources demonstrate differing levels of effectiveness, it remains unclear which source provides the best results for neuroprotection and regeneration in TON. The optimal timing and dosage for EV treatments have yet to be established, and further studies are needed to refine the protocols for EV administration. Additionally, enhancing the delivery and retention of EVs in the retina is crucial for maximizing their therapeutic potential, with biomaterials emerging as a potential solution. Extended observation periods are necessary to thoroughly assess the safety, efficacy, and long-term effects of EV therapies for TON, as most studies conducted so far have only monitored outcomes for up to 60 days. Furthermore, understanding the influence of the retinal microenvironment and glial cell activation on EV-mediated neuroprotection and neuroregeneration is essential for optimizing therapeutic strategies.

The novelty of this review lies in its comprehensive integration of recent advances in cell-based approaches for the treatment of TON, including strategies aimed at enhancing graft survival and promoting axon regeneration. While previous reviews have primarily focused on general neuroprotective strategies, this review emphasizes the latest findings on the molecular and cellular mechanisms underlying RGC replacement and regeneration. Additionally, we discuss the therapeutic potential of NSPCs for TON, highlighting their capacity to provide neuroprotective effects and serve as neuronal relays. Furthermore, this review critically evaluates the translational potential of these approaches by examining recent preclinical findings and ongoing clinical trials.

The significance of this review extends beyond TON to broader applications in optic nerve regeneration and neurodegenerative retinal diseases. The principles governing RGC survival, integration, and axonal regeneration are highly relevant to conditions such as glaucoma, optic neuritis, and ischemic optic neuropathy. By identifying key barriers to successful cell-based interventions, this review contributes to ongoing efforts to develop more effective regenerative strategies for a wide range of optic neuropathies. Furthermore, the discussion of recent advances in cell-free therapies highlights the potential for alternative strategies that circumvent the limitations of direct cell transplantation. The ability of stem cell-derived EVs to modulate neuroinflammation and enhance endogenous repair processes is a promising avenue for future research.

Future research in this field should focus on refining cell differentiation protocols to generate specific RGC subtypes with enhanced survival and functional integration properties. Advances in single-cell transcriptomics and CRISPR-based gene editing may facilitate the selective enrichment of RGC subpopulations with optimal regenerative potential. Additionally, future studies should investigate the role of the ECM and the retinal microenvironment in regulating graft-host interactions. Emerging bioengineering strategies, such as 3D bioprinting and scaffold-based transplantation, may provide novel approaches to enhance cell survival and axonal guidance. Furthermore, interdisciplinary collaborations among stem cell biologists, neuroengineers, and clinicians will be essential for translating experimental findings into clinically viable therapies.

Finally, the clinical translation of cell-based therapies for TON will require rigorous preclinical validation and the establishment of standardized protocols for cell preparation, delivery, and functional assessment. While early-phase clinical trials have demonstrated the safety of stem cell-based interventions for TON, their efficacy in restoring vision has yet to be conclusively established. Future clinical studies should incorporate advanced imaging techniques, such as functional MRI and optical coherence tomography, to objectively evaluate the functional integration of transplanted cells. Additionally, patient selection criteria, optimal timing for transplantation, and long-term safety considerations must be carefully addressed before these therapies can be widely adopted in clinical practice.

In conclusion, cell-based therapies hold immense potential for treating TON, yet several critical challenges must be addressed to achieve functional optic nerve regeneration. The integration of recent advances in stem cell biology, gene editing, and biomaterial engineering offers exciting opportunities to enhance the efficacy of these approaches. By tackling current limitations and leveraging interdisciplinary innovations, future research may pave the way for effective regenerative treatments that restore vision in patients with TON and other optic neuropathies.

## Data Availability

*Not applicable*.
